# Maintaining maximal metabolic flux by gene expression control

**DOI:** 10.1371/journal.pcbi.1006412

**Published:** 2018-09-20

**Authors:** Robert Planqué, Josephus Hulshof, Bas Teusink, Johannes C. Hendriks, Frank J. Bruggeman

**Affiliations:** 1 Department of Mathematics, Vrije Universiteit Amsterdam, Amsterdam, The Netherlands; 2 Systems Bioinformatics, Vrije Universiteit Amsterdam, Amsterdam, The Netherlands; University of Illinois at Urbana-Champaign, UNITED STATES

## Abstract

One of the marvels of biology is the phenotypic plasticity of microorganisms. It allows them to maintain high growth rates across conditions. Studies suggest that cells can express metabolic enzymes at tuned concentrations through adjustment of gene expression. The associated transcription factors are often regulated by intracellular metabolites. Here we study metabolite-mediated regulation of metabolic-gene expression that maximises metabolic fluxes across conditions. We developed an adaptive control theory, *q*ORAC (for ‘Specific Flux (q) Optimization by Robust Adaptive Control’), and illustrate it with several examples of metabolic pathways. The key feature of the theory is that it does not require knowledge of the regulatory network, only of the metabolic part. We derive that maximal metabolic flux can be maintained in the face of varying *N* environmental parameters only if the number of transcription-factor binding metabolites is at least equal to *N*. The controlling circuits appear to require simple biochemical kinetics. We conclude that microorganisms likely can achieve maximal rates in metabolic pathways, in the face of environmental changes.

## Author summary

To attain high growth rates, microorganisms need to sustain high activities of metabolic reactions. Since the catalysing enzymes are in finite supply, cells need to carefully tune their concentrations. When conditions change, cells need to adjust those concentrations. How cells maintain high metabolism rates across conditions by way of gene regulatory mechanisms and whether they can maximise metabolic activity is far from clear. Here we present a general theory that solves this metabolic control problem, which we have called *q*ORAC for specific flux (*q*) Optimisation by Robust Adaptive Control. It considers that external changes are sensed by internal “sensor” metabolites that bind to transcription factors in order to regulate enzyme-synthesis rates. We show that such a combined system of metabolism and its gene network can self-optimise its metabolic activity across conditions. We present the mathematical conditions for the required adaptive control for robust system-steering to optimal states across conditions. We provide explicit examples of such self-optimising coupled metabolism and gene network systems. We prove that a cell can be robust to changes in *K* parameters, e.g. external conditions, if at least *K* internal metabolite concentrations act transcription-factor binding sensors. We find that the optimal relation of the enzyme synthesis rates of self-optimising systems and the concentration of the sensor metabolites can generally be implemented by basic biochemistry. Our results indicate how cells are able to maintain maximal reaction rates, even in changing conditions.

## Introduction

Microbes need to grow fast to outcompete others. They therefore have to maintain high growth rates in changing environments. To achieve this specific metabolic fluxes (metabolic rates per unit of expended enzyme) need to be kept as high as possible. Since metabolic enzymes are a limited resource, cells should behave economically: synthesise the right enzymes in the right amounts, and adapt their levels when conditions change. In this paper we show how cells can achieve this in the case when the growth rate itself is fixed, but a limited protein pool needs to be optimally distributed over metabolic pathway reactions to maximise its steady-state rate.

**Fig 1:**
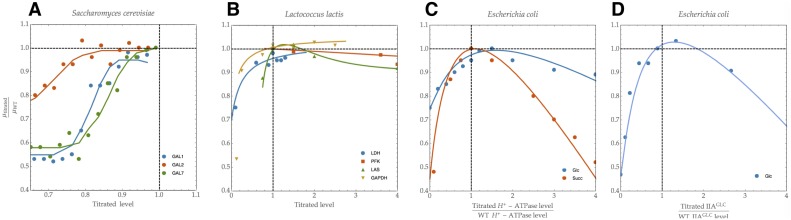
Experimental evidence indicating that microbes tune their enzyme levels to maximise growth rate. In each example, the wild type (WT) is shown to express enzyme concentrations at which the growth rate *μ* is approximately maximal. Axes show enzyme concentrations relative to wild type (WT) levels (abscissa) and growth rates relative to WT. Data adapted from: A, [9]; B, [4, 5, 6], C, [3]; D, [2]. Abbreviations: GAL1, galactokinase; GAL2, Galactose permease; GAL7, Galactose-1-phosphate uridyl transferase; LDH, lactate dehydrogenase; PFK, phosphofructokinase; LAS, las operon; GAPDH, glyceraldehyde 3-phosphate dehydrogenase; Glc, glucose; Succ, succinate. In [9] there are many other examples, including several proteins that do not show levels at which growth rate is optimised.

**Fig 2:**
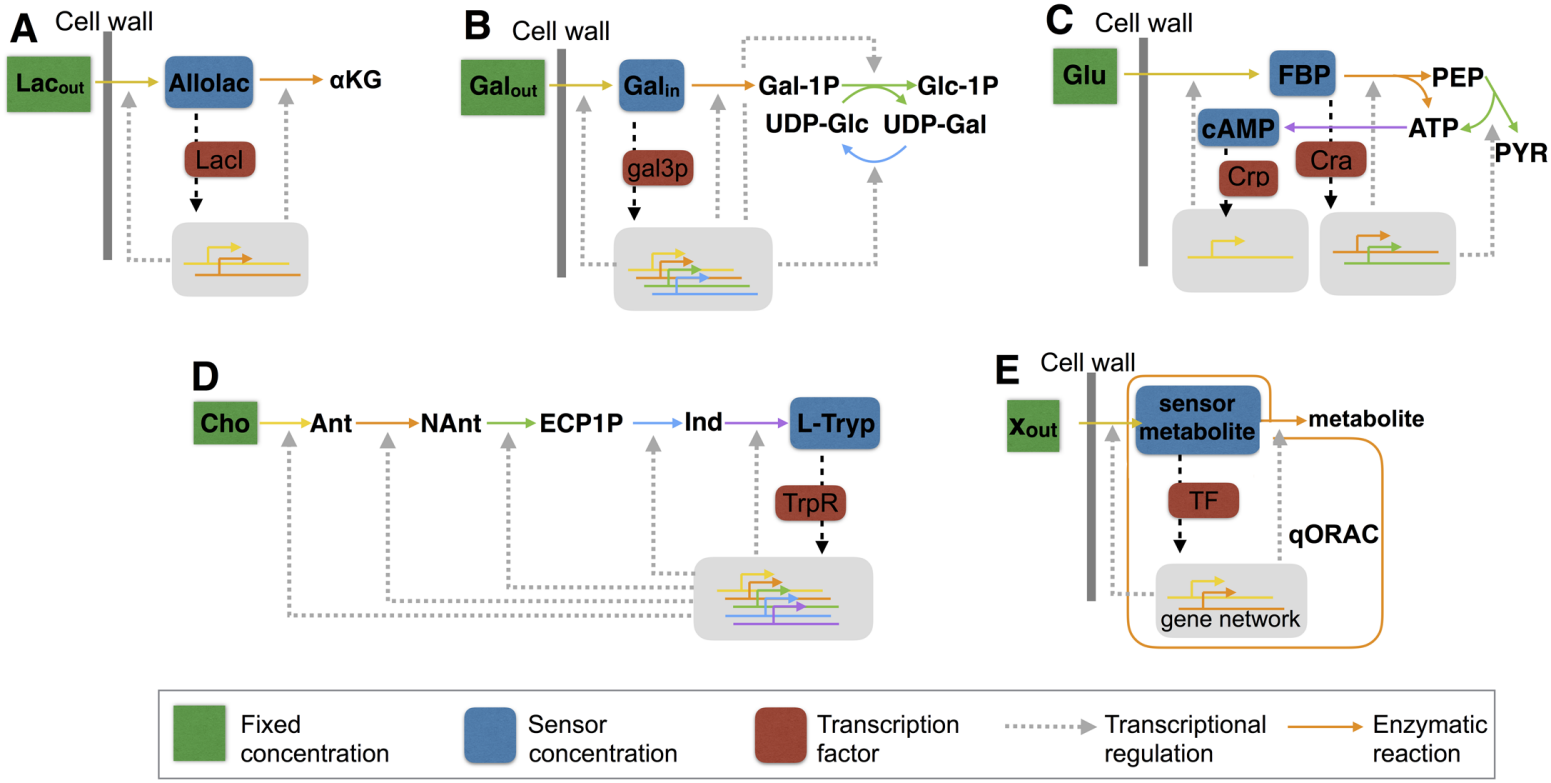
Biological examples of *q*ORAC. Four well-characterised metabolic pathways in which a metabolite binds to a transcription factor (TF) to influence gene expression. The *q*ORAC framework applies to each of them: in each case, the *q*ORAC formalism gives rise to the enzyme synthesis rates that steer the metabolic pathway to maximal metabolic rates that are robust to changes in the external concentration (external with respect to the pathway). (A) The lac operon in *E. coli*, with sensor Allolactose binding to LacI; (B) The galactose uptake system in yeast, with sensor internal galactose binding to gal3p; (C) The control of glycolytic enzymes via sensors FBP (binding to Cra), and cAMP (binding to Crp); (D) The control of the L-Tryptophan biosynthesis pathway by the amino acid binding to TrpR; (E) The general scheme of a *q*ORAC-steered pathway. Abbreviations:Lac_out_, external lactose; Allolac, allolactose; *α*KG, *α*-ketoglutarate;Gal_out_, external galactose; Gal_in_, internal galactose; Gal-1P, galactose-1-phosphate; Glc-1P, glucose-1-phosphate; UDP-Glc, uridine-diphosphate-glucose; UDP-Gal, uridine-diphosphate-galactose; Glu, glucose; FBP, fructose-1,6-biphosphate; PEP, phosphoenolpyruvate; PYR, pyruvate; cAMP, cyclic AMP; ATP, adenosine-triphosphate; Cho, chorismate; Ant, Anthranilate; NAnt, N-(5’-phosphoribosyl)-anthranilate; ECP1P, Enol-1-0-carboxy-phenylamino-1-deoxyribulose phosphate; Ind, Indole-3-glycerol-P; L-Tryp, L-tryptophan.

**Fig 3:**
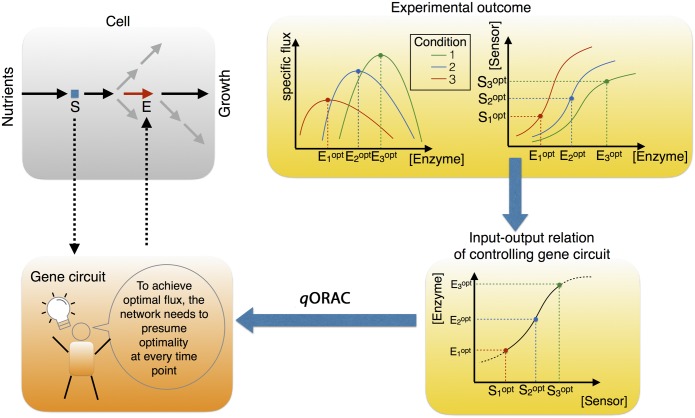
Integrating *q*ORAC with experimental evidence. Top, left: We consider a cell which takes up glucose (Glu) and converts it into biomass using a metabolic pathway. A sensor metabolite (*S*) influences gene expression and hence enzyme levels. Let *E* be one such enzyme in the active pathway. Top, right: The concentration of *E* is titrated experimentally under different glucose conditions, Low, Mid and High. In each condition, the maximal growth rate is measured, at different levels of titrated enzyme levels. In the same experiment, the sensor concentration is monitored. Bottom, right: Plotting the optimal enzyme levels at different conditions together with the measured sensor concentrations indicates the input-output relation of the gene network necessary to achieve maximal growth rates. Any gene network that implements such an input-output relation automatically expresses optimal enzyme levels in each condition. Bottom, left: To ensure that the steady state of the combined metabolic-gene system is always optimal, the gene network must presume optimality of the sensor at each time point. If the sensor is not optimal, it will change (and so will the enzyme levels); if it is optimal and stationary, the whole pathway will achieve maximal rates. *q*ORAC also decribes the input-output relation in other conditions than the cell may have experienced (dotted lines in graph bottom right).

**Fig 4:**
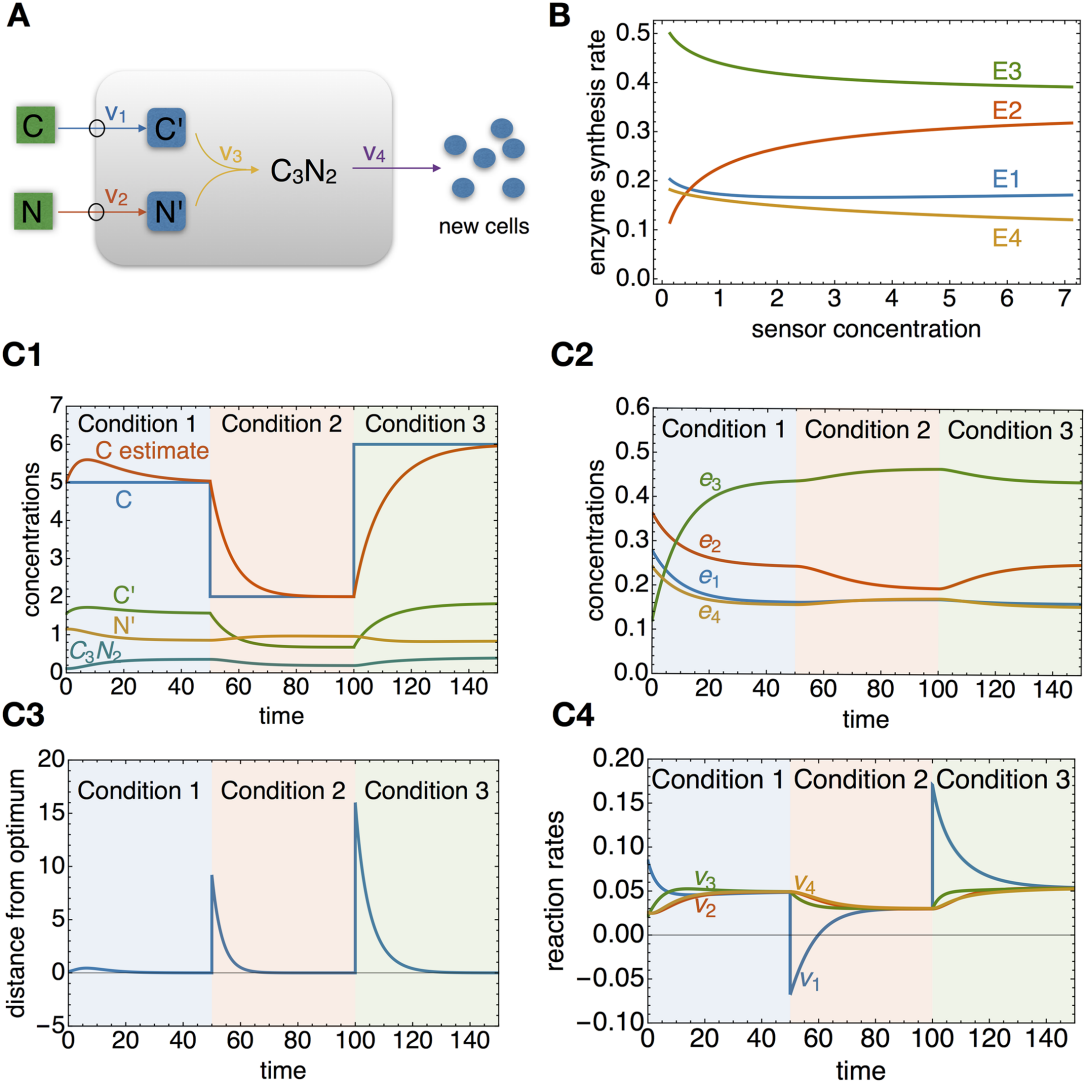
Example *q*ORAC dynamics. The dynamics are illustrated for the network shown in A. The green box depicts a varying external concentration, the blue box denotes the sensor concentration. B: the optimal input-output relations, showing enzyme synthesis rates as a function of changing sensor concentration *C*′. In plots C1 to C4, the external *C* concentration is changed after 50 time units, and again after 100 time units. C1: The optimal C concentration predicted by the sensor (red line) converges to the real external C concentration (blue), even when the external concentration changes at *t* = 50 and *t* = 100. C2: enzyme dynamics equilibrate after each change in external conditions, and reach their optimal levels. C3: the steered metabolic pathway reaches the optimum after each external change, as the distance to the (periodically changing) optimum reaches zero after some time. C4: flux dynamics equilibrate, showing that the pathway has reached steady state each time the external conditions change. Full equations are given in Box 2, code is given in the SI.

**Fig 5:**
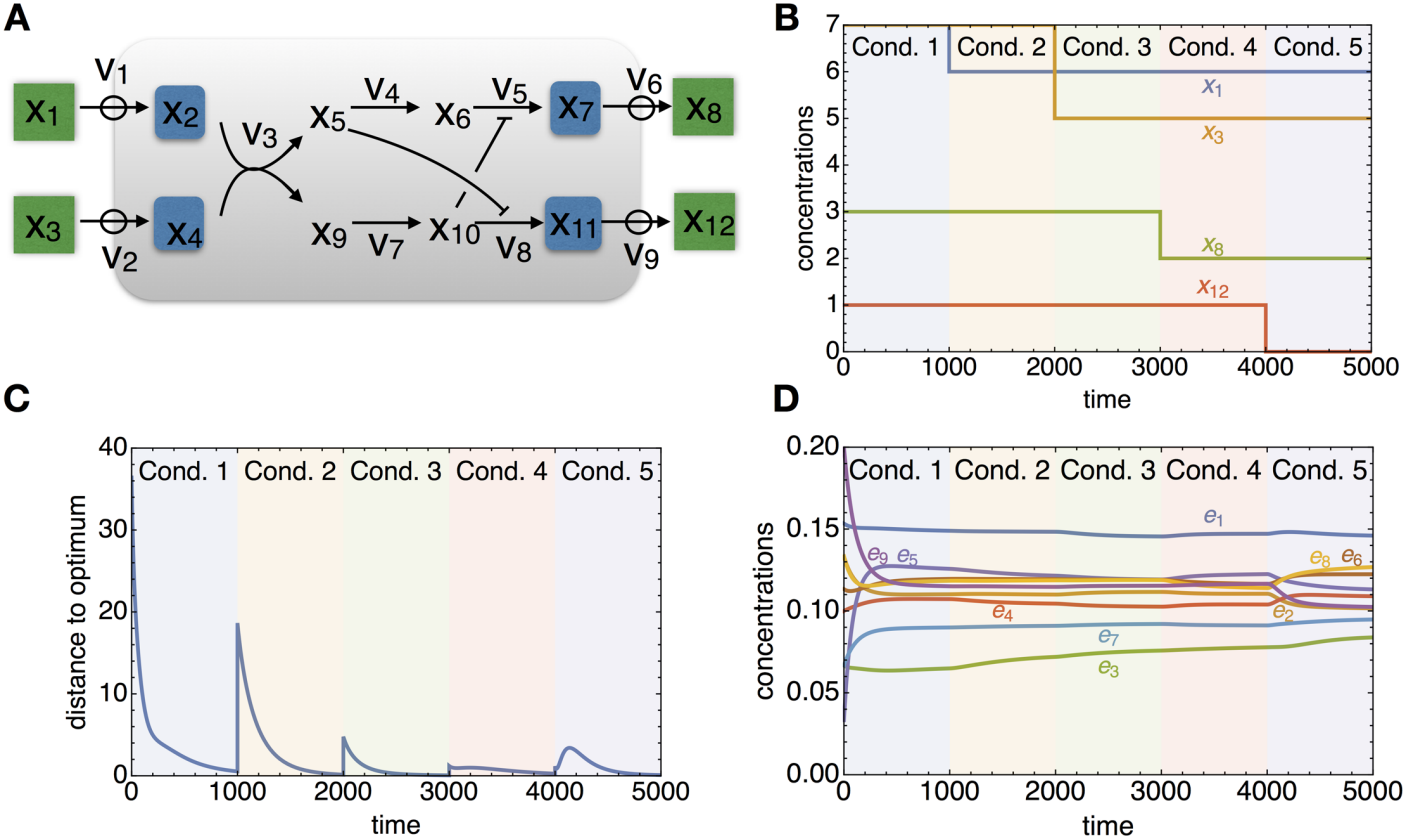
Example dynamics for a more complicated pathway. A: metabolic network with two inputs and two outputs, and with allosteric cross-inhibition. This pathway is robust to changes in both input and output concentration (in green), which requires four sensors (in blue). B: Each of the external concentrations is changed once, and the system adapts accordingly. C: the metabolite concentrations converge to the (periodically changing) predicted optimum over time. D: enzyme concentration dynamics. See SI text for details of the pathway, and the matlab file daes_double_branched.m for the code.

**Fig 6:**
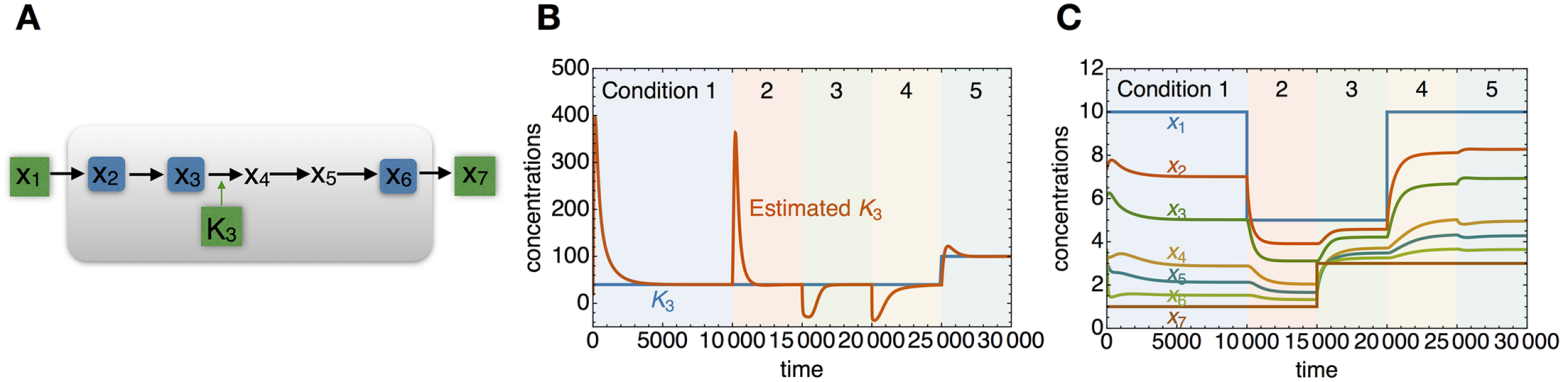
*q*ORAC for an internal parameter. In this example *q*ORAC is illustrated for a *K*_*m*_ parameter in the third reaction, *K*_3_. In A the same pathway is drawn, with sensors in blue. B: metabolite dynamics in which first external concentrations are varied (green) and at the end also *K*_3_ is varied. C: *K*_3_ (in green) is varied at time *t* = 2500, and the predicted optimal value (in orange) subsequently converges, illustrating robust adaptive control. An example in which the same pathway is controlled using a different set of sensors, resulting in lack of convergence to the optimum, is found in the SI, S4 Fig.

Experimental evidence is mounting that cells are indeed able to tune enzyme levels to maximise the growth rate (Fig 1; [1, 2, 3, 4, 5, 6, 7, 8, 10]). Efficient enzyme allocation has also recently been shown explain measured flux values [11], and to underlie a surprising number of other general physiological phenomena [12, 13], such as the bacterial growth laws [14, 15, 16], overflow metabolism (the Crabtree or Warburg effect; [13, 17]), and catabolite repression [18]. Except perhaps for the case of optimal ribosomal synthesis [15, 16], it is not clear in any of these examples how cells can find the optimal protein expression state out of all possible ones.

Finding optimal states is difficult for microorganisms. They generally do not have sensor proteins in their membranes to alert them of the presence or absence of nutrients or stresses, because their membrane space is limited. It needs to be filled with transporters and respiratory proteins that directly contribute to fitness. Thus cells have to decide how to allocate their resources from internal cues only. Cells are evidently able to accomplish this feat, but that raises the question how they are able to achieve such “blind optimisation”.

Gene expression regulation is largely achieved by transcription factors that are either affected by direct binding of metabolites, or signal transduction cascades, as readouts of environmental and cellular states. Even though transcription factor binding by sensor metabolites is widely accepted in the field [19, 20], the identity of the sensors is only known in a handful of cases (Fig 2). In *E. coli*, fructose-1,6-bisphosphate (FBP), a glycolytic intermediate, binds to the transcription factor Cra to regulate genes involved in glycolysis [18, 21]; in yeast, the galactose catabolic pathway is induced by intracellular galactose [22]; in *E. coli*, uncharged-tRNAs induce synthesis of ppGpp when amino acids are limited, leading to the adjustment of ribosome expression [15, 16]; like most most amino acid pathways, the amino acid L-tryptophan regulates the transcription of several enzymes involved in its own biosynthetic pathway [23]; perhaps the best known example is the lactose operon, which is induced by allolactose, an intermediate of the pathway [24]. There is even very recent experimental evidence that *E. coli*’s central metabolism is in fact controlled by just three such sensor metabolites (cyclic AMP (cAMP), FBP and fructose-1-phosphate (F1P); [25]).

What remains unexplained is why certain sensor metabolites bind to transcription factors and others do not. How many sensors can we expect to be functioning? When do cells rely on just a few sensors? What are the design criteria for regulating circuits that maintain optimal metabolism in fluctuating environments? Does this regulation require complex, hard to evolve, biochemistry or it is almost gratuit? We derive a universal theory, called *q*ORAC (for Specific Flux (*q*) Optimisation by Robust Adaptive Control), that gives answers to these questions. Understanding how growth rate itself is maximised is beyond the scope of this paper. Instead we focus on the important case of maximising specific rates of metabolic subnetworks at fixed growth rate.

In order to achieve maximal metabolic rates without *direct* knowledge of those external conditions and how they change, a controlling gene regulatory network must work as follows. At each point in time, internal sensor metabolites must influence a gene regulatory network, causing changes in gene expression. The strength of this signal depends on the concentration of the sensor metabolite. The crucial ingredient is that the gene network must be made in such a way that it expresses proteins at optimal steady state rates with respect to the current sensor metabolite concentration. The network thus ‘assumes’ a steady-state optimum at each point in time. As long as there is a mismatch between the enzyme synthesis rates and the external concentration, so that the metabolic system is not in an optimal state, the system will display dynamics. The sensor metabolite concentration will therefore continue to change and the enzyme synthesis rates will change with it. However, when the enzyme rates are optimal, given the current external environment, a steady state should be reached, which is then necessarily also optimal. In this way, the cell has achieved an optimal state without direct information about it. Its allocation of limited biosynthetic resources for protein synthesis will then be optimal. A gene network, informed by sensor metabolites, that causes optimal steady state enzyme levels in different conditions therefore must necessarily implement some form of *q*ORAC control (Fig 3).

The experimental evidence presented in Fig 1 indicates that a *q*ORAC-like control mechanism is active in cells. If cells are able to reach maximal growth rates (and hence maximal metabolic rates to attain this) in different conditions, at different optimal enzyme concentrations, then the gene regulatory network responsible must necessarily cause the correct enzyme synthesis rates (or must approximate these to a good degree). If this gene network works on the basis of internal metabolic information (rather than on information from signalling pathways, for instance), the control is adaptive, and indeed a form of *q*ORAC control.

Remarkably, the *q*ORAC theory we present here shows that a metabolic system, coupled to its controlling gene network, has a unique optimal steady state, no matter what the environmental conditions are—even though that optimum *changes* with those conditions. We prove that the dynamics of enzyme synthesis that is required for attaining optimal metabolic states can be inferred from the kinetic rate laws from metabolic enzymes alone. This is in direct agreement with a celebrated engineering principle, the internal model principle [26]. Our results also suggest that the optimising enzyme dynamics of a gene circuits circuit can be achieved with basic biochemistry.

The *q*ORAC theory predicts which metabolites may act as sensors. A fundamental insight is that maintenance of optimal metabolism in the face of *N* parameters requires *N* sensor metabolites. The *q*ORAC theory indicates that recent findings, such as the pervasive optimisation of enzyme levels in yeast [9], or the small number of sensor metabolites found in *E. coli*’s central metabolism [25], should necessarily be seen as surprising.

The phenotypic plasticity of microorganisms is a marvel of evolution. What would be even more remarkable is that cells can maximise their performance in changing conditions, without direct information about those changes. This appears almost impossible in view of the bewildering biochemical complexity of the cell. Part of what we achieve in this paper is to show that this skepticism is most likely unfounded: cells can do this. The insight can explain the robustness to human interventions in metabolic engineering and medicine, and provide opportunities to circuit design in synthetic biology.

### Motivating *q*ORAC with an example

We will first introduce the control problem that a cell faces. We consider a well-understood example: the regulation of galactose metabolism in yeast (Figs 1A and 2B). We aim to characterise the dynamics of a controlling gene circuit that always maximises the steady-state flux per unit invested enzyme in this pathway (the *specific flux*) upon an environmental change, such as in the extracellular galactose concentration. The controlling gene network has to distribute a finite amount of biosynthetic resources for enzyme synthesis over the four pathway enzymes to maximise the steady state pathway flux.

Depending on the external galactose concentration, less or more enzymatic resources should be invested in the galactose import reaction. This leaves a correspondingly smaller or larger pool of enzymatic resources for the remaining pathway reactions. An increase in [Gal_out_] will cause an increase in [Gal_in_], which is therefore indicative for the external change. Gal_in_ can thus act as a signal for the adjustment of enzyme concentrations of the pathway: the transporter concentration should decrease and the others should increase.

In yeast, Gal_in_ plays the role of metabolic sensor [27]. It relays information to the GAL operon by binding to gal3p, a regulatory protein that can activate transcription factors, such as gal80 and gal4. The key question is how the concentration of Gal_in_ should influence the gene network in order to steer the galactose pathway to maximal specific flux.

We refer to the relation between the steady-state concentrations of the metabolic sensor ([Gal_in_]) and the metabolic enzymes as the input-output relation of the gene circuit. *q*ORAC specifies this relation for robust maximisation of specific pathway flux. Whether a gene circuit with realistic biochemical kinetics can be found that can implement this input-output relation then still needs to be determined. Since the gene network for the galactose pathway in yeast is known, the optimal input-output relation may be found by fitting parameters in this network, which we achieved in an earlier paper [28]. In the current paper, however, we show that the problem of finding optimal input-output relations for a given metabolic pathway has a general solution, applicable to all examples shown in Fig 2A–2D. This indicates that cells can implement *q*ORAC using simple regulating circuits.

## Methods

The *q*ORAC theory starts with the dynamics of the intracellular metabolite concentrations ***x***_*I*_ = (*x*_1_, …, *x*_*n*_) of a metabolic network,

$$\begin{array}{c} \dot{x_{I}}=Nv\left(x_{I};x_{E}\right)-\mu x_{I}. \end{array}$$ (1)

Here, *N* is the stoichiometry matrix, ***v***(***x***_*I*_; ***x***_*E*_) is the vector of reaction rates, ***x***_*E*_ are fixed external concentrations, and *μ* is the cellular growth rate. It is generally assumed that the dilution rate of concentrations by growth, −*μ****x***_*I*_, is negligible for metabolism. We take the same view here, and consider

$$\begin{array}{c} \dot{x_{I}}=Nv\left(x_{I};x_{E}\right). \end{array}$$ (2)

The *q*ORAC framework couples this metabolic pathway to enzyme dynamics, by choosing

$$\begin{array}{c} \dot{e}=E\left(x_{S}\right)-\mu e. \end{array}$$ (3)

Since enzyme dynamics occur at time scales of similar order as the growth rate, the dilution by growth cannot be neglected this time. Throughout the paper, the growth rate is a predefined parameter, and not part of the optimisation problem (see the Discussion for more information). ***E***(***x***_*S*_) denote the enzyme synthesis rates for all the different enzymes involved in the pathway. These functions may only depend on internal sensor metabolite concentrations, as explained in the Introduction. The task is to define these functions in such a way that the combined dynamical metabolic-enzyme system converges to a steady state in which flux through the pathway is maximal.

## Results

As explained in the Introduction, *q*ORAC relies on allocating resources on the basis of sensor metabolite information alone. The optimal allocation must therefore be uniquely defined for each set of sensor concentrations. By considering the optimisation problem in detail, we show that this requires several steps:

1. We must restrict the original pathway to a minimal set of metabolic reactions, connecting sources to sinks, called an Elementary Flux Mode (EFM). EFMs have been shown to arise naturally within the context of this optimisation problem [29, 30], as we will see.
2. Having restricted the pathway to an EFM, the optimisation problem of maximal steady state specific flux must have, for each choice of external conditions, a unique optimal allocation of enzymes and internal metabolites. We show that for practically all enzyme kinetics rate laws, this is the case.
3. We show under what conditions a set of chosen sensor concentrations may be used in a *q*ORAC control. One of the conditions is that the number of sensors must be equal to the number of varying external concentrations.
4. The metabolic pathway, coupled to *q*ORAC enzyme dynamics, must have a unique steady state, which is necessarily optimal. We show this is true for a very large class of pathways.

We now consider these four steps in detail.

### Step 1: Restricting to minimal pathways

We aim to maximise a steady-state specific flux *v*_*r*_/*e*_*T*_ through the network where *v*_*r*_ is some chosen output flux (e.g. in mM/hr) and *e*_*T*_ (e.g. in grams) is total amount of invested enzyme. The optimisation problem we study is

$$\begin{array}{c} \max_{x_{I},e}\{\frac{v_{r}}{e_{T}}|\;Nv=0,\;\sum_{j}e_{j}=e_{T}\}, \end{array}$$ (4)

with *e*_*j*_ as the concentration of enzyme *j*. Thus, we wish to maximise a given output flux *v*_*r*_ per unit of total invested enzyme *e*_*T*_ of a metabolic network at steady state.

The optimisation problem stated in Eq (4) is equivalent to minimising the amount of enzyme necessary to sustain a given steady-state flux *v*_*r*_ at rate *V*_*r*_,

$$\begin{array}{c} \min_{x_{I},e}\{\frac{\sum_{j}e_{j}}{v_{r}}\;|\;Nv=0,\;v_{r}=V_{r}\}. \end{array}$$ (5)

A crucial observation is now that since reaction functions generally are of the form *v*_*j*_ = *e*_*j*_*f*_*j*_(***x***_*I*_; ***x***_*E*_) [31], we may prescribe *v*_*r*_ = 1. After all, if we can solve that problem then we can solve it for *v*_*r*_ = *V*_*r*_ as well by multiplying all the enzyme concentrations by *V*_*r*_, because the specific flux *v*_*r*_/*e*_*T*_ remains the same. Hence, we simplify (5) to

$$\begin{array}{c} \min_{x_{I},e}\{\sum_{j}e_{j}\;|\;Nv=0,\;v_{r}=1\}. \end{array}$$ (6)

The relation *v*_*j*_ = *e*_*j*_*f*_*j*_(***x***_*I*_; ***x***_*E*_) may also be used to write *e*_*j*_ = *v*_*j*_/*f*_*j*_(***x***_*I*_; ***x***_*E*_) and rewrite (6) to

$$\begin{array}{c} \min_{x_{I}}\{\sum_{j}\frac{v_{j}}{f_{j}\left(x_{I};x_{E}\right)}\;|\;Nv=0,v_{r}=1\}. \end{array}$$ (7)

Observe that the enzyme concentration vector ***e*** has disappeared from the problem. (Note also that this optimisation is not a stoichiometric-model optimisation, such as flux balance analysis [32]. The *q*ORAC method takes into account the kinetics of the metabolic enzymes and the metabolite concentrations are the variables in this approach. The outcome of *q*ORAC is the definition of a self-optimising dynamical system; this has nothing to do with the optimisation associated with stoichiometric modelling.)

It has recently been shown that the flux profiles that solve (7) (and therefore also the equivalent original problem (4)) are always subnetworks with a particularly simple structure, called Elementary Flux Modes (EFMs; [30, 29]). Such EFMs are one-degree-of-freedom flux vectors satisfying *N****v*** = 0 that cannot be simplified further by deleting reactions without violating the steady state assumption [33, 34]. A given EFM is thus characterised by λ(*V*_1_, …, *V*_*m*_), where λ is a free parameter and the flux vector (*V*_1_, …, *V*_*m*_) is fixed.

### Step 2: Unique allocation within a given EFM

If we want to optimise specific flux *within a given EFM* with flux vector (*V*_1_ …, *V*_*m*_), we still need to find a vector ***x***_*I*_ for

$$\begin{array}{c} \min_{x_{I}}\{\sum_{j}\frac{\lambda V_{j}}{f_{j}\left(x_{I};x_{E}\right)}\;\}. \end{array}$$ (8)

This motivates the introduction of the *objective function*

$$\begin{array}{c} O\left(x_{I}\right):=\sum_{j}\frac{\lambda V_{j}}{f_{j}\left(x_{I};x_{E}\right)}, \end{array}$$ (9)

which is to be minimised, for given external concentrations ***x***_*E*_, by suitably choosing internal concentrations ***x***_*I*_. This function is convex for pathways with many kinds of reaction kinetics [11], and in the Supporting Information (SI) we show that it is in fact strictly convex, for an even larger class of rate laws. Hence, the optimum is uniquely specified by the external concentrations ***x***_*E*_.

Note that the objective function has a lower value if the values of *f*_*j*_(***x***_*I*_; ***x***_*E*_) are higher. Maximising specific flux may thus be reinterpreted as maximising the values of all *f*_*j*_’s simultaneously. These *f*_*j*_ are closely associated to the saturation levels of enzyme *j* with its reactants (and effectors). This optimisation can be done by making as little enzyme as possible, so that the enzymes are used at their maximal capacity.

If we find the vector $x_{I}^{o}$ which minimises *O*(***x***_*I*_), then we can infer the corresponding optimal enzyme concentrations ***e***^*o*^ by setting

$$\begin{array}{c} e_{j}^{o}=\frac{\lambda V_{j}}{f_{j}\left(x_{I}^{o};x_{E}\right)}. \end{array}$$ (10)

It is clear that we may choose λ = 1 in *O*(***x***_*I*_): having found the minimiser of *O*(***x***_*I*_) for λ = 1, we have found it for all λ: the corresponding enzyme levels $e_{j}^{o}$ just scale with λ. In hindsight, we may also for instance normalise the enzyme concentrations such that they sum to a given total concentration *e*_*T*_.

### Step 3: Implementing *q*ORAC: Choosing the right (number of) sensors

At this stage, the optimal enzyme concentrations that maximise the specific flux at steady state are still defined in terms of external concentrations ***x***_*E*_: for each choice of ***x***_*E*_, the objective function (9) needs to be minimised to find $x_{I}^{o}$, and subsequently ***e***^*o*^ needs to be calculated. In order to characterise gene regulatory networks that produce the right concentrations of enzymes in steady state, robustly with respect to changes in external concentrations but without direct knowledge of those changes, we need to understand the defining characteristics of optimal solutions.

Steady-state optimisers $x_{I}^{o}$ are minima of *O*(***x***_*I*_), and are dependent on (i.e., parameterised by) ***x***_*E*_. So, $x_{I}^{o}$ is a (in fact, the) critical point of *O*(***x***_*I*_) = *O*(*x*_1_, …, *x*_*n*_), satisfying the *optimality relations*

$$\begin{array}{c} 0=\frac{\partial O}{\partial x_{i}}=\frac{\partial }{\partial x_{i}}\sum_{j}\frac{V_{j}}{f_{j}\left(x_{I};x_{E}\right)},\;i=1,\ldots ,n. \end{array}$$ (11)

So instead of minimizing *O*(***x***) for given external conditions ***x***_*E*_, we could solve (11) by prescribing ***x***_*E*_ and solving for the remaining variables, the internal concentrations ***x***_*I*_. However, the gene network does not have access to ***x***_*E*_. Eq (11) should be solved with knowledge of the current sensor concentrations only. We therefore solve (11) by prescribing a subset of the internal metabolite concentrations, sensor values ***x***_*S*_, and solving for all remaining concentrations, namely all other internal concentrations, but now also the (unknown) external concentrations. The solution is denoted by ***ξ*** = (***ξ***_*I*_, ***ξ***_*E*_), and is the estimated optimal concentration vector, under the assumption of steady state and optimality of the sensor values. In short, we call ***ξ*** the optimum as predicted by the sensors. Here, ***ξ***_*E*_ are the external concentrations for which the current sensor values would have been optimal if the pathway had been in steady state. The part of ***ξ***_*I*_ corresponding to sensor metabolites, ***ξ***_*S*_, of course coincides with the real concentrations ***x***_*S*_, by construction. Since ***ξ*** is defined by ***x***_*S*_, we denote it by ***ξ***(***x***_*S*_).

To solution of ∂*O*(***x***)/∂*x*_*i*_ = 0 for different sensor values is well-defined mathematically if the Implicit Function Theorem (IFT) holds (see SI for a more detailed exposition). In essence, this means that it is then possible to calculate the optimal allocation by varying the sensors appropriately. The sensors are able to “track” the optima. Any choice of sensor metabolites for which the IFT holds is a candidate for the proposed adaptive control. An immediate consequence of the IFT is that the number of sensor metabolite concentrations must equal the number of changing external metabolite concentrations to which the system needs to be robust. This makes intuitive sense: to track changes (and hence achieve robustness) in *N* parameters, the gene network should be influenced by (at least) *N* (independent) internal sensors. Examples of parameters are environmental nutrient concentrations, temperature, pH and toxin concentrations.

### Step 4: The *q*ORAC pathway has a unique steady state, the optimum

With ***ξ***(***x***_*S*_), we can define corresponding predicted optimal enzyme levels, analogous to (10), by setting

$$\begin{array}{c} e_{j}^{o}=\frac{V_{j}}{f_{j}\left(\xi \left(x_{S}\right)\right)}. \end{array}$$ (12)

At these enzyme concentrations, the pathway is either in steady state or not. If not, the metabolic concentrations are still changing, including the sensor concentrations. Hence, the predicted optimal enzyme levels also change. This argument indicates that the only steady state of the metabolic network steered in this fashion is the optimal one.

In the SI we prove that an EFM metabolic pathway with added *q*ORAC control has a unique steady state, the optimum. The proof is fully worked out for linear chains of enzymatic reactions (Theorem 3 in SI), but the techniques of the proof extend to a much larger class of pathways. All one needs to require is that for each choice of enzyme concentrations, the metabolic pathway has a unique steady state (a common enough assumption), and that the sensors are a few reaction steps away from the external concentrations (which makes intuitive sense). This result therefore ensures that when the *q*ORAC-controlled pathway has reached a steady state, it necessarily must be optimal.

### Putting it all together

We now finish by implementing the enzyme synthesis rate functions *E*_*j*_ in

$$\begin{array}{c} {\dot{e}}_{j}=E_{j}-\mu e_{j}. \end{array}$$

By setting

$$\begin{array}{c} E_{j}=\mu \frac{V_{j}}{f_{j}\left(\xi \left(x_{S}\right)\right)}, \end{array}$$ (13)

we have ensured that at steady state the enzyme levels are optimal. The complete construction is termed *q*ORAC, and is summarised in Definition 1. A fully-worked out example for the small pathway shown in Figs 3 and S2 is specified in Example 1.

**Definition 1 (*q*ORAC):** The following differential-algebraic system of equations implements Specific Flux (*q*) Optimisation by Robust Adaptive Control (*q*ORAC) through an EFM with flux vector (*V*_1_, …, *V*_*m*_) in a cell culture growing at fixed growth rate *μ*. Let *I* be the index set of internal metabolite concentrations, *E* the index set of external concentrations, and *S* the index set of sensor concentrations. Let furthermore $O\left(x_{I}\right)=\sum_{j=1}^{m}V_{j}/f_{j}\left(x_{I};x_{E}\right)$ be the objective function. Then we consider for *i* ∈ *I*, and *j* = 1, …, *m*,

$$\begin{array}{c} {\dot{x}}_{i}=\sum_{j=1}^{m}N_{ij}v_{j}=\sum_{j=1}^{m}N_{ij}e_{j}f_{j}\left(x_{I};x_{E}\right), \end{array}$$ (14)

$$\begin{array}{c} {\dot{e}}_{j}=E_{j}\left(x_{S}\right)-\mu e_{j}, \end{array}$$ (15)

$$\begin{array}{c} E_{j}\left(x_{S}\right)=\mu \frac{V_{j}/f_{j}\left(\xi \left(x_{S}\right)\right)}{\sum_{l=1}^{m}V_{l}/f_{l}\left(\xi \left(x_{S}\right)\right)}, \end{array}$$ (16)

where ***ξ***(***x***_*S*_) = (***ξ***_*I*_(***x***_*S*_), ***ξ***_*E*_(***x***_*S*_)) is the predicted optimum, and is the (time-dependent) solution of

$$\xi_{S}=x_{S},$$ (17)

$$\frac{\partial O}{\partial \xi_{i}}\left(\xi \right)=0.$$ (18)

The rescaling of *E*_*j*_(***x***_*S*_) in (16) by the sum of all the inverses of 1/*f*_*j*_ implies that total enzyme concentration is chosen to be equal to 1. Other rescalings give identical results, up to the chosen scaling factor. The choice above, however, is particularly useful, since it ensures positive synthesis rates both for positive and negative metabolic rates through the pathway, and it ensures that it is well-defined also at thermodynamic equilibrium (see SI for details).

**Example 1: *q*ORAC for a simple pathway** The example *q*ORAC-controlled metabolic pathway from Figs 4 and S2 is specified by the following set of equations for the metabolite concentrations ***x*** = (*x*_1_, …, *x*_4_) = ([*C*], [*C*′], [*N*′], [*C*_3_*N*_2_]). Note that *x*_1_ = [*C*] is an external concentration which may change value periodically, as shown in Fig 4.

$$\begin{array}{l} {\dot{x}}_{1}=0,\\ {\dot{x}}_{2}=v_{1}-v_{3},\\ {\dot{x}}_{3}=v_{2}-v_{3},\\ {\dot{x}}_{4}=v_{3}-v_{4}, \end{array}$$

where *v*_*i*_ = *e*_*i*_*f*_*i*_(***x***), *i* = 1, …, 4, and the kinetics functions *f*_*i*_(***x***) are defined by

$$\begin{array}{l} f_{1}\left(x\right)=\frac{0.6x_{1}-0.75x_{2}}{\left(0.2x_{1}+1.0\right)\left(0.33x_{2}+1.0\right)},\\ f_{2}\left(x\right)=\frac{\left[N\right]-3.0x_{3}}{\left(0.5x_{3}+1\right)\left(\left[N\right]+1\right)},\\ f_{3}\left(x\right)=\frac{0.2x_{2}x_{3}-0.17x_{4}}{\left(0.2x_{3}/5+1.0\right)\left(0.33x_{2}+0.17x_{4}+1.0\right)},\\ f_{4}\left(x\right)=\frac{x_{4}-0.0025}{0.33x_{4}+1.0025}. \end{array}$$

The objective function is given by $O\left(x\right)=\frac{1}{f_{1}\left(x\right)}+\cdots +\frac{1}{f_{4}\left(x\right)}$. The enzyme dynamics are given ${\dot{e}}_{j}=E_{j}\left(x_{2}\right)-e_{j}$, *j* = 1, … 4, where

$$\begin{array}{c} E_{j}\left(x_{2}\right)=\frac{1/f_{j}\left(\xi \left(x_{2}\right)\right)}{\sum_{k}1/f_{k}\left(\xi \left(x_{2}\right)\right)},\;j=1,\ldots ,4 \end{array}$$

and the predicted optimum ***ξ***(*x*_2_) is defined by

$$\begin{array}{c} \left\{ \begin{array}{ccc} \xi_{2} & = & x_{2},\\ \frac{\partial O}{\partial \xi_{2}}\left(\xi_{1},\ldots ,\xi_{4}\right) & = & \frac{\partial O}{\partial \xi_{3}}\left(\xi_{1},\ldots ,\xi_{4}\right)=\frac{\partial O}{\partial \xi_{4}}\left(\xi_{1},\ldots ,\xi_{4}\right)=0. \end{array}\right. \end{array}$$

### Illustrations of the *q*ORAC framework

A toy metabolic network, with two external parameters and one output flux, is shown in Fig 4 (see Box 2 for the mathematical implementation). In this example, only the external [C] concentration is allowed to vary, so one internal sensor metabolite is required. Upon changes in this external concentration, the sensor concentration changes, causing changes in enzyme synthesis, which finally result in adaptation to the new optimum. The optimal enzyme synthesis relations of the gene network are also shown. They are simple curves, suggesting that small gene circuits are sufficient for optimal steering of this pathway.

To illustrate the general applicability of *q*ORAC, consider the complicated branched example network in Fig 5. It has two inputs and two outputs and two allosteric interactions; by employing four sensors, it can be made robust to changes in all four external concentrations.

The *q*ORAC framework is able to start from nearly any initial condition. As an extreme example, with no enzymes present, and only the sensor concentration and no other internal metabolite, the *q*ORAC-controlled pathway still steers to optimum (S1 Fig). Similarly, if the sensor concentrations are ‘wrong’, such that they predict a metabolic flow in the opposite direction to the one dictated by external concentrations, the combined controlled system nevertheless converges to the correct optimum (S2 Fig).

The *q*ORAC control does not guarantee that a metabolic pathway is actually steered towards the optimum. In an example in which one of the periodically changing parameters is a *K*_*m*_ parameter of a rate law, the choice of sensors matters critically (Figs 6 and S3). With one choice, the system robustly steers to the optimal specific flux steady state, but with another choice it does not. In both cases, the technical requirements to use the internal metabolites as sensors are met.

### Biological examples

In each of the pathways shown in Fig 2A–2D, the sensor metabolite(s) and transcription factor(s) have been identified. Specifying the kinetics for each enzymatic step in the pathway now directly gives the corresponding objective function (9) and the *q*ORAC framework can be set up. The case of galactose uptake (Fig 2B) in yeast has been studied theoretically in detail by [28], including fitting the parameters of the well-characterised GAL gene network to approximate optimal input-output relations. Recent experimental evidence moreover shows that yeast cells are indeed able to tune the levels of these enzymes to optimise growth rate ([9]; Fig 1A).

## Discussion

Experimental evidence is accumulating that suggests that cells can tune their enzyme resources to maximise growth rate [1, 2, 3, 4, 5, 6, 7, 8, 10]. We addressed whether cells growing at a fixed rate can tune limited enzyme resources to steer metabolism to optimal flux states, given only limited information about the current metabolic state of the cell in the form of sensor-metabolite concentrations. We demanded robustness of optimality in the face of environmental changes. We logically derived the *q*ORAC framework, which implements such control for Elementary Flux Modes, the minimal steady state pathways that maximise specific flux [29, 30]. Maximisation of specific fluxes is a requirement for maximisation of the specific growth rate of cells.

We use the term Specific Flux (*q*) Optimisation by Robust Adaptive Control (*q*ORAC) to describe the regulatory mechanism that we study. ‘Robust’ signifies that attaining optimal states is independent of (environmental) parameter values—the system is robust to them. ‘Adaptive’ means that the control system steers the metabolic system to optimality without direct knowledge of external changes, contrary to the more widely studied problem of ‘optimal control’, in which the steering mechanism works using external changes as inputs to the controller [35].

It is important to note that the growth rate itself is not optimised in our approach. Maximising steady state growth rate rather than specific flux requires a fundamentally different approach. The modelling framework should be extended to Metabolite-Enzyme models in which enzymes are made from precursors [36, 37]. In such models, the growth rate features quadratically rather than linearly, in the resulting steady state and optimality equations. EFMs therefore no longer apply, and the objective function *O*(***x***) is also absent. Our approach is therefore more suitable to isolated pathways then to all of metabolism. For such smaller pathways, it is more reasonable to assume that there is a fixed amount of enzyme resources to distribute, and that the cellular growth rate is considered constant. Recent work does suggest, however, that the objective function *O*(***x***) studied here in fact matters to cells also on a more global metabolic level [11].

An important finding of our work is that the number of sensor metabolites must be (at least) equal to the number of parameters for which the metabolic pathway is robustly optimal. In other words, if the metabolic pathway always achieves states of maximal specific flux, regardless of the values of three (independently changing) environmental parameters, such as, for example, osmolarity, temperature and some nutrient concentration, then the number of sensors is expected to be three. This is a general result that follows from the associated mathematics of this control problem. Finding the sensors experimentally is difficult, and the number of known sensors is still quite small. However, it is telling that the whole of central carbon metabolism in *E. coli* seems to be controlled by just three sensors, FBP, cAMP and F1P [25].

The identity of suitable sensors does not follow immediately from the optimisation problem. In general, one needs to make sure that the Implicit Function Theorem applies to the optimum Eq (11), and this is not a trivial matter. However, a different argument shows that sensors near the beginning or ends of the pathway would work in most cases. The reason is that for all metabolites in between a set of fixed concentrations, their optimal value is uniquely determined by minimising the corresponding optimisation problem (i.e. finding the minimum of a suitable objective function *O*(***x***; ***x***_*S*_) with ***x*** the set of metabolites between the sensors ***x***_*S*_). The remaining variables, including the external concentrations, then need to be determined using the optimum Eq (11). This is easiest (it involves the smallest number of equations and unknowns to solve for) when sensors are close to the external metabolites. Also from a biological standpoint this makes sense: such sensors obviously provide the most information of any change in external concentrations.

An important question is whether the adaptive control can be achieved by molecular circuits, given our understanding of biochemical kinetics and molecular interactions. The explicit example from galactose metabolism in yeast [28] gives hope that this might be true in general. If the necessary gene network is small, then the optimal circuit is likely also evolvable. We cannot give definite answers about this, but the computational analyses of different networks, of which some are shown in this paper, indicate that *q*ORAC-controlled networks show remarkably simple dynamics and input-output relations. One would expect that biochemical systems are capable of evolving those, and that synthetic biologists are capable of designing them.

The parameterisation of the optimising circuit is completely determined by the kinetics and the wiring of the metabolic pathway that it controls, since the objective function (9) contains only this information. This interdependence between the controller and the controlled is sometimes called the ‘internal model principle’ in engineering [26] which roughly states that the control system should have knowledge of the dynamic behaviour of the system in order to be able to control it. Additional control mechanisms may then prevent for instance undesired oscillations or slow responses.

The internal model principle, applied to metabolic pathway control, suggests a new perspective on the larger problem of understanding metabolic regulation. The theory presented here indicates that knowledge of the metabolic pathway, including properties of catalysing enzymes, is sufficient to understand how this pathway needs to be controlled to maximise flux. It is not necessary to know the controlling regulatory pathway in advance. This offers hope for situations in which this circuit has not been characterised yet, or for which it needs to be designed synthetically.

Technological advances have spurred recent interest in studying control properties of gene regulatory networks in cellular metabolism. One line of work involves characterising a particular gene control system and studying its theoretical properties. Examples are the perfect adaptation in the chemotaxis network in *E. coli* [38, 39], the robustness properties of the heat-shock response system [40] and of the circadian clock [41]. Several authors have considered dynamic optimisation of resources in pathways from a mostly computational perspective, e.g. to minimise the time of adaptive response [42], deFBA [43], and for other objectives than maximal specific flux, such as detecting equilibrium regimes of pathways [44], robustness to flux perturbations [45], and noise propagation [46]. In many studies, the control is not adaptive, but optimal; the objective is then usually to maximise the long term production of biomass [47, 42, 48, e.g.].

The approach taken here differs principally from most previous works in the following respect. The objective (maximal specific flux) is defined in advance, and the optimal input-output relations are characterised later. The framework is also analytic rather than computational: the input-output relations are obtained by solving the optimum equations (11) for the pathway, rather than by using a numerical optimisation routine. The latter is impossible, since this would require knowing the external concentrations.

A few recent papers have used adaptive controls similar to ours. So-called Flux Control Regulation (FCR; [49]) comes closest, and uses the same type of adaptive control as proposed in *q*ORAC. FCR also explicitly relies on making estimates at each time point under the assumption of steady state. When the system is in fact in steady state, it has reached the desired objective. The principle difference between FCR and *q*ORAC lies in the objective. The input-output relations in FCR come from measurements and ensure steady state properties only. *q*ORAC, however, solves a steady state optimisation problem, and constructs input-output relations directly from the kinetic rate laws of the metabolic pathway itself. Another recent example of a coarse-grained model of cellular physiology including gene expression control can be found in [50]. Two other examples using adaptive control are from the context of optimal ribosomal allocation to maximise the growth rate in *E. coli*. The free amino acid concentration acts as a sensor to ppGpp, which downstream influences gene expression. Two models have been proposed that are based on optimal synthesis of ribosomes so as to maximise growth rate [15, 16]. The input-output relations used in these models are not derived from kinetic properties as in *q*ORAC, but are designed by hand to approximate maximal growth rates in different conditions.

The choice of sensors sometimes matters for the control to steer the pathway to optimum (Figs 6 and S4). This example already indicates that, although the *q*ORAC control follows logically from the design objective, it is not easy to decide which intermediate metabolites make it controllable. We cannot expect completely general mathematical theorems. Apparently, some choices of sensors do work, and others do not, for the same pathway, using the same initial conditions. A second, mathematical reason why one cannot expect convergence to optimal states is that if time would be reversed, the control would remain the same, but dynamics would be reversed. The control is based on steady state properties of the system, and these do not change upon time reversal.

*q*ORAC has direct applications in synthetic biology. To achieve maximal production rates in a biotechnological-product producing pathway requires a controller that *q*ORAC provides. The only ingredient to design such a controller are the enzymatic rate laws in the pathway. *q*ORAC then immediately makes predictions about the optimal enzyme synthesis rates, as a function of one or more intermediate metabolites. As the synthetic biology field advances, synthetic circuits with the required input-output relationships for the constituent enzymes of the pathway can be designed and built. *q*ORAC therefore does not only contribute to the general understanding of steering mechanisms to optimal states, but provides direct operational relevance for microbiology, synthetic biology and biotechnological applications.

## Supporting information

S1 Text Supporting information text in which we prove that the optimisation problem (8) has a unique solution for a large class of reaction kinetics. We also give a detailed explanation which and how many sensor metabolites may be used in *q*ORAC. We prove that many pathways with *q*ORAC control only have one steady state, the actual optimum. We also give additional illustrations of the *q*ORAC formalism, give details on the numerical integration of *q*ORAC-controlled pathways, and fully describe the kinetics of the pathways considered in this paper.
(PDF)

S1 Fig Extreme robustness: A simulation for the same pathway as in Fig 3 in the main text, but now with minimal initial conditions: At the start, enzymes are completely absent, and all internal metabolites except the sensor are absent. The pathway is still steered to the optimal specific flux steady state. A: the pathway; B: metabolite concentrations over time (all except orange), and predicted optimal external metabolite concentration (orange); C: reaction fluxes over time; D: enzyme concentrations over time. See Box 2 in main text for details of the pathway, and the matlab file daes_CN_minimal_ICs.m for the code.
(TIF)

S2 Fig With initial conditions for the sensor concentration such that they actually predict an optimal flow from end to beginning rather than the reverse, the predicted optimum needs to ‘straighten out’, and move through a singular point: Thermodynamic equilibrium. Although the requirements for sensor control are not upheld in this point, the predicted optimum moves smoothly through this singular points and the system adapts as it should. A: linear chain pathway, with external conditions such that flow is initially from *x*_1_ to *x*_7_; B: dynamics for the predicted optimal metabolite concentrations (***ξ***). The intersection point of all the curves is thermodynamic equilibrium; C: reaction flux dynamics. Note that the fluxes do not pass through *v*_1_ = ⋯ = *v*_6_ = 0. Three do, and the others do not, in this example. See SI text for details of the pathway, and the matlab file daes_linearchain_reversal.m for the code.
(TIF)

S3 Fig An example of qORAC steering for an changing internal parameter, showing lack of convergence to the optimum. A: The pathway, which is identical to that in Fig 5 in the main text—only the choice of sensors (in blue) is different. Sensor *x*_3_ is swapped with *x*_4_. B/C: The dynamics of metabolites (B) and predicted *K*_3_ values (C) do start to change. However, the dynamics converge to a singular point, and the dynamical system can not continue. This second choice of sensors does not yield a gene expression control system which steers the pathway to optimal specific flux. See matlab code daes_extra_param_wrong.m for the code.
(TIF)

S4 Fig King-Altman patterns of an ordered *M* − *K* mechanism. The left one shows the master pattern, the middle and right figure show two alternative patterns that yield the constant term in the denominator of *f*.
(TIF)
