# Supplementary material for: Maintaining maximal metabolic flux by gene expression control
Source: PLoS Comput Biol. 2018 Sep 20;14(9):e1006412. doi: 10.1371/journal.pcbi.1006412 (PMC6168163; doi:10.1371/journal.pcbi.1006412)
Supplement: S1 Text — We also give a detailed explanation which and how many sensor metabolites may be used in qORAC. We prove that many pathways with qORAC control only have one steady state, the actual optimum. We also give additional illustrations of the qORAC formalism, give details on the numerical integration of qORAC-controlled pathways, and fully describe the kinetics of the pathways considered in this paper. (PDF) [file pcbi.1006412.s001.pdf]

Supporting Information for "Maintaining maximal metabolic flux by gene expression control"

R. Planqué, J. Hulshof, B. Teusink, J. C. Hendriks and F. J. Bruggeman

August 29, 2018

Uniqueness of the optimum at fixed external concentrations

In the qORAC framework the sensors are used to predict an optimum of the objective function by following a starting critical point. It is therefore vital that this optimum is unique. If the objective function would have multiple critical points, the steering mechanism could end up in a local minimum and not steer towards the global optimal specific flux steady state. Here we prove uniqueness of the optimum for a wide class of possible reaction rate functions, thereby showing that for each chosen set of external concentrations, the objective function only has one critical point: the global optimum. The reconstructed predicted optimum by the sensors thus coincides with this global optimum if the sensor concentrations have the right values (the ones from the global optimum defined by the external concentrations).

The objective function $O(\boldsymbol{x}) = \sum_{j=1}^{m} \frac{V_j}{f_j(\boldsymbol{x})}$ has recently been shown to be convex in logarithmic variables $y_i = \log x_i$ for a particular choice of widely used enzyme kinetics rate laws, called "convenience kinetics" [1], rate laws of the form

$$f(\boldsymbol{x}) = \frac{\prod_p x_p^{r^p} - K_{eq} \prod_q^N x_q^{r_q}}{S(\boldsymbol{x})},$$

where $S(\boldsymbol{x})$ is a polynomial of the concentrations with strictly positive coefficients. Here we improve on this result and show that the objective function is in fact strict convex. For the optimisation problem we need to consider only its behaviour on the relevant metabolite concentration domain $C := \{(x_1, \dots, x_N) \mid f_j(\boldsymbol{x}) \geq 0 \ j = 1, \dots, m\}$. This is generally a convex and compact set [1]. The functions $1/f_j(\boldsymbol{x})$ become unbounded where the kinetics functions $f_j(\boldsymbol{x})$ approach zero, i.e., near the boundary of the domain C. A strict convex function on a compact bounded domain has a unique minimum, thus making the optimisation problem well-posed.

We show that each function in the sum is strict convex in its arguments. We illustrate the main argument for reversible Michaelis-Menten kinetics,

$$f(x_1, x_2) = \frac{x_1 - K_{eq} x_2}{a + b x_1 + c x_2},$$

Then, writing $y_i = \log x_i$, and using that $x_1 > K_{eq} x_2 > 0$,

$$\begin{aligned}
\frac{1}{f(x_1, x_2)} &= \frac{a + b x_1 + c x_2}{x_1 - K_{eq} x_2} \\
&= \frac{1}{x_1} \frac{a + b x_1 + c x_2}{1 - K_{eq} \frac{x_2}{x_1}} \\
&= \frac{1}{x_1} (a + b x_1 + c x_2) \sum_{n=0}^{\infty} \left(K_{eq} \frac{x_2}{x_1} \right)^n \\
&= e^{-y_1} (a + b e^{y_1} + c e^{y_2}) \sum_{n=0}^{\infty} \left(K_{eq} e^{y_2 - y_1} \right)^n
\end{aligned}$$

This is a sum of strict convex functions, and hence is strict convex. Transforming back to original variables leaves the strict convexity invariant. If each term in the objective function is strict convex, then $O(\boldsymbol{x})$ is strict convex as well in the directions spanned by all the individual reactions' directions. The only property that therefore needs to be checked is that $O(\boldsymbol{x})$ is strict convex in all directions. Below we give a detailed exposition of strict convexity of the inverse rate laws for a number of enzyme mechanisms, such as Ordered and Random mechanisms with multiple substrates and products, the Thorell-Chance mechanism, and the Ping-Pong mechanism. Inhibition and activation are also easily treated in all of these cases. We also provide one counter example, the Botts-Morales mechanism [2], which is shown not to have a strict convex inverse rate law. (This does not imply that it must have multiple minima, only that we cannot conclude uniqueness from convexity.) We finally note that the method using the geometric series is not applicable in all cases, such as in cooperative kinetics. Such cooperative rate laws do appear to give strict convex inverse rate laws, however.

The more complicated mechanisms we discuss all have multiple substrates and multiple products. To obtain the rate laws from reaction kinetics, the graphical method of King-Alman is used [3]. We will introduce this method briefly the first time we use it, in the proof of Ordered mechanisms. For a full explanation and examples of this method we refer to Cornish-Bowden [4, §4.2] or the original paper [3]. The King-Altman is a method with which one can give an exhaustive list of all the terms in the numerator and especially the denominator of a rate law. As we have seen in the proof of strict convexity of the reversible Michaelis-Menten scheme, it suffices to show that $1/f$ is strict in enough directions. The King-Altman method allows us to pick and choose enough terms in the denominator to conclude strict convexity in all directions.

Ordered mechanism

We use nomenclature as introduced by Cleland [5]. In an ordered mechanism the substrates are bound to the enzymes in some fixed order, and products are released in fixed order. As an example, the following scheme represents the Ordered Bi-Bi mechanism:

$$E \overset{\overset{S_1}{\downarrow}}{\longleftrightarrow} ES_1 \overset{\overset{S_2}{\downarrow}}{\longleftrightarrow} ES_1S_2/EP_1P_2 \overset{\overset{P_1}{\uparrow}}{\longleftrightarrow} EP_2 \overset{\overset{P_2}{\uparrow}}{\longleftrightarrow} E,$$

For the general case, we consider the reaction

$$\sum_{i=1}^{M} S_i \longleftrightarrow \sum_{j=1}^{K} P_j,$$

which we will call an $M-K$ mechanism.

Theorem 1. *An Ordered $M-K$ mechanism has strict convex function $1/\tilde{f}$ in all variables $\sigma_i, i = 1, \ldots, M$ and $\pi_j, j = 1, \ldots, K$.*

Proof. The case where $M = K = 1$ is considered as a special case and is already proven above. For $M \geq 2, K \geq 2$ we take certain terms in the denominator D of f and we will prove that these terms give enough directions to make $1/\tilde{f}$ strict convex. For our analysis we use that the denominator D of f contains the terms

$$1, s_1, s_1s_2, \ldots, s_1s_2\cdots s_M, p_K, p_Kp_{K-1}, \ldots, p_Kp_{K-1}\cdots p_1.$$

There are many other terms in D, but in this proof we show that the terms above give enough directions for strict convexity of $1/\tilde{f}$. First we will show by analysis of the King-Altman patterns that these terms actually appear in the denominator of f. The master pattern is shown in Figure S4, which is the skeleton of the scheme. The King-Altman method finds patterns that 1) consists of lines from the master pattern, 2) connects every pair of enzyme forms and 3) contains no closed loops [3]. This means the number of enzyme forms in every pattern is one more than the number of lines. Per enzyme form, per pattern, we

draw arrowheads in the patterns, such that where-ever in the scheme you start, the pattern leads to this enzyme form. The final terms that end up in the denominator consist of all variables that are added in the reactions that align with the arrowheaded pattern. Each arrowheaded pattern leading to an enzyme gives a denominator term in the rate equation. For the proof and examples of this method we refer to [3] or [4, §4.2]. The King-Altman patterns can be interpreted as the patterns that you get when you walk towards an enzyme complex, e.g. $ES_1S_2 \dots S_\alpha$ in the master scheme when one reaction S_α in the mechanism is blocked. The notation that we will use in this chapter for this pattern is $ES_1S_2 \dots S_\alpha(S_\alpha)$. For our mechanism, the arrowheaded pattern that yields the constant term in D can be obtained in two different ways, as shown in Figure S4. In the middle figure we walk towards enzyme complex E, while the reaction that adds P_1 is blocked. We will use notation $E(P_1)$ for this pattern. The right figure shows an alternative way to obtain the constant denominator term. We walk towards E, while blocking the reaction where S_M is added, we denote this as $E(S_M)$.

Using the notation introduced above, the terms in the denominator of f that we will use are obtained by the following King-Altman patterns:

$$
\begin{aligned}
1 &: E(P_1) \text{ or } E(S_M),\\
s_1 &: ES_1(P_1),\\
s_1s_2 &: ES_1S_2(P_1),\\
&\vdots\\
s_1s_2\dots s_M &: ES_1S_2\dots S_M(P_1),\\
p_K &: EP_K(S_M),\\
p_Kp_{K-1} &: EP_KP_{K-1}(S_M),\\
&\vdots\\
p_Kp_{K-1}\dots p_1 &: EP_KP_{K-1}\dots P_1(S_M).
\end{aligned}
$$

To conclude this proof, we first write $1/\tilde{f}$ in logarithmic variables, in the same way as before. Let f be given by

$$
\begin{aligned}
f &= \frac{\prod_{i=1}^{M} s_i - \frac{1}{K_{eq}} \prod_{j=1}^{K} p_j}{D},\\
D &= K_1 + K_{s_1}s_1 + K_{s_{12}}s_1s_2 + \dots\\
&\quad + K_{s_{12\cdots M}}s_1s_2\cdots s_M + K_{p_K}p_K +\\
&\quad + K_{p_{KK-1}}p_Kp_{K-1} + \dots + K_{p_{KK-1\cdots 1}}p_Kp_{K-1}\cdots p_1\\
&\quad \text{and other terms.}
\end{aligned}
$$

Note that the K_is in this way of writing the rate equation are not Michaelis-Menten constants. The relation of K_i with Michaelis-Menten constants for small mechanisms is given by [5]. Using the same rewriting procedure as in the reversible Michaelis-Menten example, we find

$$
\begin{aligned}
1/f &= D\Big(\frac{1}{\prod_{i=1}^{M} s_i} \sum_{n=0}^{\infty} \frac{1}{K_{eq}^n} \Big(\frac{\prod_{j=1}^{K} p_j}{\prod_{i=1}^{M} s_i}\Big)^n\Big),\\
1/\tilde{f} &= \tilde{D}\Big(e^{-\sum_{i=1}^{M}\sigma_i} \sum_{n=0}^{\infty} \frac{1}{K_{eq}^n} e^{n(\sum_{j=1}^{K}\pi_j - \sum_{i=1}^{M}\sigma_i)}\Big),\\
\tilde{D} &= K_1 + K_{s_1}e^{\sigma_1} + K_{s_{12}}e^{\sigma_1+\sigma_2} + \dots\\
&\quad + K_{s_{12\cdots M}}e^{\sigma_1+\sigma_2+\dots+\sigma_M}\\
&\quad + K_{p_K}e^{\pi_K} + K_{p_{KK-1}}e^{\pi_K+\pi_{K-1}} + \dots\\
&\quad + K_{p_{KK-1\cdots 1}}e^{\pi_K+\pi_{K-1}+\dots+\pi_1} \text{ and other terms,}
\end{aligned}
$$

where we used transformation $s_i \mapsto e^{\sigma_i}, i = 1, \ldots, M, p_j \mapsto e^{\pi_j}, j = 1, \ldots, K$. We will now give directions in which $1/\tilde{f}$ is strict convex. The above equation for $1/\tilde{f}$ gives strict convexity of $1/\tilde{f}$ in directions $\boldsymbol{r} \in \text{span}\{\boldsymbol{a}_i + \boldsymbol{v}_1 + n\ \boldsymbol{v}_2,\ i = 1, 2, \ldots\}$, where $\boldsymbol{a}_i$ are the columns of matrix $A = (\boldsymbol{a}_1, \boldsymbol{a}_2, \ldots)$. This matrix is produced by the denominator part of $1/\tilde{f}$. Vectors $\boldsymbol{v}_1$ and $\boldsymbol{v}_2$ are given by the numerator part of $1/\tilde{f}$.

$$\boldsymbol{r} = \left(\begin{array}{c} \sigma_1 \\ \sigma_2 \\ \sigma_3 \\ \vdots \\ \sigma_M \\ \hline \pi_1 \\ \pi_2 \\ \pi_3 \\ \vdots \\ \pi_{K-2} \\ \pi_{K-1} \\ \pi_K \end{array}\right), \quad A = \left(\begin{array}{c|cccc|cccc} 0 & 1 & 1 & \cdots & 1 & 0 & 0 & \cdots & 0 \\ 0 & 0 & 1 & & 1 & 0 & 0 & & 0 \\ 0 & 0 & 0 & & 1 & 0 & 0 & & 0 \\ \vdots & & & & & & & & \\ 0 & 0 & 0 & & 1 & 0 & 0 & & 0 \\ \hline 0 & 0 & 0 & & 0 & 0 & 0 & & 1 \\ 0 & 0 & 0 & & 0 & 0 & 0 & & 1 \\ 0 & 0 & 0 & & 0 & 0 & 0 & & 1 \\ \vdots & & & & & & & & \\ 0 & 0 & 0 & & 0 & 0 & 0 & & 1 \\ 0 & 0 & 0 & & 0 & 0 & 1 & & 1 \\ 0 & 0 & 0 & & 0 & 1 & 1 & & 1 \end{array}\right),$$

and

$$\boldsymbol{v}_1 = \left(\begin{array}{c} -1 \\ -1 \\ -1 \\ \vdots \\ -1 \\ \hline 0 \\ 0 \\ 0 \\ \vdots \\ 0 \\ 0 \\ 0 \end{array}\right), \quad \boldsymbol{v}_2 = \left(\begin{array}{c} -1 \\ -1 \\ -1 \\ \vdots \\ 1 \\ \hline 1 \\ 1 \\ 1 \\ \vdots \\ 1 \\ 1 \\ 1 \end{array}\right).$$

In this case we will consider only $n = 0$, since this will give enough directions of strict convexity. We get strict convexity of $1/\tilde{f}$ in $\boldsymbol{r} \in \text{span}\{\boldsymbol{a}_i + v_1, i = 1, 2, \ldots\} = \text{span}\{\boldsymbol{b}_i, i = 1, 2, \ldots\}$, with

$$B = (\boldsymbol{b}_1, \boldsymbol{b}_2, \dots) = \left(\begin{array}{c|cccc|cccc}
-1 & 0 & 0 & \cdots & 0 & -1 & -1 & \cdots & -1 \\
-1 & -1 & 0 & & 0 & -1 & -1 & & -1 \\
-1 & -1 & -1 & & 0 & -1 & -1 & & -1 \\
\vdots & & & & & & & & \\
-1 & -1 & -1 & & 0 & -1 & -1 & & -1 \\
\hline
0 & 0 & 0 & & 0 & 1 & 1 & & 1 \\
0 & 0 & 0 & & 0 & 0 & 1 & & 1 \\
0 & 0 & 0 & & 0 & 0 & 0 & & 1 \\
\vdots & & & & & & & & \\
0 & 0 & 0 & & 0 & 0 & 0 & & 1 \\
0 & 0 & 0 & & 0 & 0 & 0 & & 1 \\
0 & 0 & 0 & & 0 & 0 & 0 & & 1
\end{array}\right).$$

This gives us a matrix that has rank $M+K$, and therefore $1/\tilde{f}$ is strict convex in $M+K$ linearly independent directions. □

Linear inhibition on an Ordered mechanism

The same procedure can also be followed to analyze linear inhibition on an Ordered mechanism. A substance is an inhibitor of a reaction if the reaction rate v decreases when the inhibitor concentration i increases, i.e. $\partial v/\partial i < 0$. The easiest kind of inhibition is product inhibition. In this section, we will analyze linear inhibition on an Ordered $M-K$ mechanism. An inhibitor binds to an enzyme complex to make this enzyme complex inactive. An inhibitor can theoretically inhibit every enzyme complex in the mechanism. Also, mixed inhibition can appear, when one inhibitor inhibits multiple enzyme complexes. Moreover, multiple inhibitors can inhibit a mechanism. The normal biological interpretation of inhibition is that it alters Michaelis-Menten constants V and K_M. Linear inhibition alters these constants in a linear way. Now we will consider one inhibitor that inhibits one enzyme complex.

Lemma 1. *If one linear inhibitor I inhibits a single enzyme complex of an $M-K$ Ordered mechanism, the function $1/\tilde{f}$ is strict convex in the variables 1,σ_1,σ_2,$\dots$,σ_M, π_1, π_2,$\dots$,π_K, ι.*

Proof. We will make a distinction between an inhibitor that inhibits a substrate complex and one that inhibits a product complex. First, we consider the case where a substrate-enzyme complex $ES_1, S_2 \dots S_\alpha, 1 \leq \alpha \leq M$ is inhibited and an inactive enzyme complex $ES_1, S_2 \dots S_\alpha I$ is formed. The denominator of f has all terms in the normal Ordered mechanism plus some extra terms, of which we use the term $s_1 s_2 \dots s_\alpha i$. The King-Altman pattern that yields this extra term is $ES_1S_2 \dots S_\alpha I(P_1)$, using the same notation as in the proof of the Ordered mechanism. The inhibitor gives one extra variable ι, with $i = e^\iota$, in f. This means that every vector in the span of columns of the matrix B is one row larger. The extra denominator term $s_1 s_2 \dots s_\alpha i$ gives an extra vector in the span; this vector, for $n = 0$, is

$$\left(\begin{array}{c} 1 \\ \vdots \\ 1 \\ 0 \\ \vdots \\ 0 \\ \hline 0 \\ \vdots \\ 0 \\ \hline 1 \end{array}\right) + \left(\begin{array}{c} -1 \\ \vdots \\ -1 \\ -1 \\ \vdots \\ -1 \\ \hline 0 \\ \vdots \\ 0 \\ \hline 0 \end{array}\right) + n \left(\begin{array}{c} -1 \\ \vdots \\ -1 \\ -1 \\ \vdots \\ -1 \\ \hline 1 \\ \vdots \\ 1 \\ \hline 0 \end{array}\right) \overset{n=0}{=} \left(\begin{array}{c} 0 \\ \vdots \\ 0 \\ -1 \\ \vdots \\ -1 \\ \hline 0 \\ \vdots \\ 0 \\ \hline 1 \end{array}\right).$$

This vector gives an extra linear independent direction of strict convexity of $1/\tilde{f}$. Therefore if I inhibits $ES_1, S_2 \dots S_\alpha$ we have a strict convex function $1/\tilde{f}$ in $1, \sigma_1, \sigma_2, \dots, \sigma_M, \pi_1, \pi_2, \dots, \pi_K, \iota$.

If I inhibits product-enzyme complex $EP_\beta P_{\beta-1} \dots P_1, 1 \leq \beta \leq K$ it will form the inactive complex $EP_\beta P_{\beta-1} \dots P_1 I$. A similar reasoning as described in the substrate-enzyme complex inhibition can be applied here. We use the extra denominator term $p_\beta p_{\beta-1} \dots p_1 i$, which is obtain by the King-Altman pattern $EP_\beta P_{\beta-1} \dots P_1 I(S_M)$. This means there is one more vector in the span of columns of B, and all vectors in the span are one row larger. The extra vector in the span, for $n = 0$, is

$$\left(\begin{array}{c} 0 \\ \vdots \\ 0 \\ \hline 0 \\ \vdots \\ 0 \\ 1 \\ 1 \\ \vdots \\ 1 \\ \hline 1 \end{array}\right) + \left(\begin{array}{c} -1 \\ \vdots \\ -1 \\ \hline 0 \\ \vdots \\ 0 \\ 0 \\ 0 \\ \vdots \\ 0 \\ \hline 0 \end{array}\right) + n \left(\begin{array}{c} -1 \\ \vdots \\ -1 \\ \hline 1 \\ \vdots \\ 1 \\ 1 \\ 1 \\ \vdots \\ 1 \\ \hline 0 \end{array}\right) \stackrel{n=0}{=} \left(\begin{array}{c} -1 \\ \vdots \\ -1 \\ \hline 0 \\ \vdots \\ 0 \\ 1 \\ 1 \\ \vdots \\ 1 \\ \hline 1 \end{array}\right).$$

Matrix B with the extra column added has rank $M + K + 1$. Therefore $1/\tilde{f}$ is a strict convex function in $1, \sigma_1, \sigma_2, \dots, \sigma_M, \pi_1, \pi_2, \dots, \pi_K, \iota$.

Finally there is the special case where I inhibits the free enzyme E and forms inactive enzyme complex EI. This case can be considered as a special case of any of the two described cases above. □

Two corollaries follow from the lemma above. First there can be mixed inhibition, where one inhibitor I inhibits multiple enzyme complexes. However, we saw that inhibition on any of the enzyme complexes gave an extra direction of strict convexity of $1/\tilde{f}$. Therefore inhibition on multiple enzyme complexes will give additional terms in the denominator of f. Second, we considered so far one inhibitor that inhibits a mechanism. It is also possible that multiple inhibitors $I_1, I_2, \dots, I_n$ inhibit a mechanism. The simplest case appears when we assume that these inhibitors operate exclusively. This means that no more than one enzyme form inhibits an enzyme complex. In this case strict convexity follows as a corollary from the lemma above, since we saw that inhibition on any enzyme complex gave strict convexity of $1/\tilde{f}$ in the direction of the inhibitor.

Thorell-Chance mechanism

The second mechanism we will consider is the Thorell-Chance mechanism. This mechanism does not contain a central complex [5]. The reaction scheme for a Bi-Bi Thorell-Chance mechanism looks like

$$E \overset{A \downarrow}{\longleftrightarrow} EA \overset{B \searrow \; P \nearrow}{\longleftrightarrow} EQ \overset{Q \uparrow}{\longleftrightarrow} E.$$

Lemma 2. *An $M - K$ Thorell-Chance mechanism has strict convex function $1/\tilde{f}$.*

Proof. As before, consider the Uni-Uni mechanism as special case. Let $M \geq 2, K \geq 2$. The function f of this mechanism has, among others, denominator terms

$$1, s_1, s_1 s_2, s_1 s_2, \dots, s_1 s_2 \cdots s_M,$$
$$p_K, p_K p_{K-1}, \dots, p_K p_{K-1} \cdots p_1,$$

Which are the same terms as we used in the case of the Ordered mechanism. However, the King-Altman patters are a little different:

$$
\begin{aligned}
1 &: E(P_1, S_M) \\
s_1 &: ES_1(P_1, S_M), \\
s_1 s_2 &: ES_1 S_2(P_1, S_M), \\
&\vdots \\
s_1 s_2 \dots s_{M-1} &: ES_1 S_2 \dots S_{M-1}(P_1, S_M), \\
s_1 s_2 \dots s_M &: EP_K P_{K-1} \dots P_2(P_2), \\
p_K &: EP_K(P_1, S_M), \\
p_K p_{K-1} &: EP_K P_{K-1}(P_1, S_M), \\
&\vdots \\
p_K p_{K-1} \dots p_2 &: EP_K P_{K-1} \dots P_2(P_1, S_M), \\
p_K p_{K-1} \dots p_1 &: ES_1 S_2 \dots S_{M-1}(S_{M-1}),
\end{aligned}
$$

where we refer to the reaction $EP_K P_{K-1} \dots P_2 \overset{S_M \searrow \quad \nearrow P_1}{\longleftrightarrow} ES_1 S_2 \dots S_{M-1}$ as (P_1, S_M). Special emphasis should be given to the two denominator terms with all substrates and all products. To conclude this proof we have to show that the terms above yield enough directions of strict convexity for $1/\tilde{f}$. The argument is identical to the one given in the proof of the Ordered mechanism. □

With very little extra work, one can prove once more that linear inhibition in a Thorell-Chance mechanism again leads to strict convex $1/\tilde{f}$.

Ping-Pong Mechanism

Our method can also be applied to Ping-Pong mechanisms. In this mechanism every substrate is converted into a product one-by-one. A Ping-Pong Bi-Bi mechanism looks like

$$E \overset{\overset{A}{\downarrow}}{\longleftrightarrow} EA/EP \overset{\overset{P}{\uparrow}}{\longleftrightarrow} E \overset{\overset{B}{\downarrow}}{\longleftrightarrow} EB/EQ \overset{\overset{Q}{\uparrow}}{\longleftrightarrow} E.$$

This mechanism is also known as a substituted-enzyme mechanism [4, 7.3.3]. Note that for a Ping-Pong mechanism the number of substrates is equal to the number of products.

Lemma 3. *An $M - M$ Ping-Pong mechanism has strict convex function $1/\tilde{f}$.*

Proof. We consider the Uni-Uni mechanism as special case. Let us consider $M \geq 2$. The function f of this mechanism does not contain all terms of the Ordered mechanism. The Ping-Pong mechanism can therefore not be considered as an extension of the Ordered mechanism. For this mechanism we consider the following

denominator term and their King-Altman patterns

$$\begin{aligned}
s_2 s_3 \dots s_M &: E_1(S_1),\\
s_1 s_3 \dots s_M &: E_2(S_2),\\
s_1 s_2 s_4 \dots s_M &: E_3(S_3),\\
&\vdots\\
s_1 s_2 \dots s_{M-1} &: E_M(S_M),\\
p_2 p_3 \dots p_M &: E_2(P_1),\\
p_1 p_3 \dots p_M &: E_3(P_2),\\
p_1 p_2 p_4 \dots p_M &: E_4(P_3),\\
&\vdots\\
p_1 p_2 \dots p_{M-1} &: E_1(P_M).
\end{aligned}$$

These terms yield enough directions to prove strict convexity of $1/\tilde{f}$. The function $1/\tilde{f}$ is strict convex in directions $\boldsymbol{r} \in \text{span}\{\boldsymbol{a}_i + \boldsymbol{v}_1 + n\boldsymbol{v}_2, i = 1, 2, \dots\}$, with matrix $A = (\boldsymbol{a}_1, \boldsymbol{a}_2, \dots)$. A is produced by the denominator part of f, $\boldsymbol{v}_1$, $\boldsymbol{v}_2$ are produced by the numerator part of f. This gives

$$\boldsymbol{r} = \left(\begin{array}{c} \sigma_1 \\ \sigma_2 \\ \sigma_3 \\ \vdots \\ \sigma_M \\ \hline \pi_1 \\ \pi_2 \\ \pi_3 \\ \vdots \\ \pi_M \end{array}\right), \quad A = \left(\begin{array}{ccccc|ccccc} 0 & 1 & 1 & \cdots & 1 & 0 & 0 & 0 & \cdots & 0 \\ 1 & 0 & 1 & & 1 & 0 & 0 & 0 & & 0 \\ 1 & 1 & 0 & & 1 & 0 & 0 & 0 & & 0 \\ \vdots & & & & & & & & & \\ 1 & 1 & 1 & & 0 & 0 & 0 & 0 & & 0 \\ \hline 0 & 0 & 0 & & 0 & 0 & 1 & 1 & & 1 \\ 0 & 0 & 0 & & 0 & 1 & 0 & 1 & & 1 \\ 0 & 0 & 0 & & 0 & 1 & 1 & 0 & & 1 \\ \vdots & & & & & & & & & \\ 0 & 0 & 0 & & 0 & 1 & 1 & 1 & & 0 \end{array}\right),$$

$$\boldsymbol{v}_1 = \left(\begin{array}{c} -1 \\ -1 \\ -1 \\ \vdots \\ -1 \\ \hline 0 \\ 0 \\ 0 \\ \vdots \\ 0 \end{array}\right), \quad \boldsymbol{v}_2 = \left(\begin{array}{c} -1 \\ -1 \\ -1 \\ \vdots \\ -1 \\ \hline 1 \\ 1 \\ 1 \\ \vdots \\ 1 \end{array}\right).$$

If we consider $n = 0$, $1/\tilde{f}$ is strict convex in directions $\boldsymbol{r} \in \text{span}\{\boldsymbol{a}_i + \boldsymbol{v}_1, i = 1, 2, \dots\} = \text{span}\{\boldsymbol{b}_i, i = 1, 2, \dots\}$,

with

$$B = (\boldsymbol{b}_1, \boldsymbol{b}_2, \dots) = \left(\begin{array}{ccccc|ccccc} -1 & 0 & 0 & \cdots & 0 & -1 & -1 & -1 & \cdots & -1 \\ 0 & -1 & 0 & & 0 & -1 & -1 & -1 & & -1 \\ 0 & 0 & -1 & & 0 & -1 & -1 & -1 & & -1 \\ \vdots & & & & & & & & & \\ 0 & 0 & 0 & & -1 & -1 & -1 & -1 & & -1 \\ \hline 0 & 0 & 0 & & 0 & 0 & 1 & 0 & & 1 \\ 0 & 0 & 0 & & 0 & 1 & 0 & 1 & & 1 \\ 0 & 0 & 0 & & 0 & 1 & 1 & 1 & & 1 \\ \vdots & & & & & & & & & \\ 0 & 0 & 0 & & 0 & 1 & 1 & 1 & & 0 \end{array}\right).$$

This matrix has rank $M+M$, which gives enough directions such that $1/\tilde{f}$ is strict convex in all its variables. □

We can again extend this to include linear inhibition.

Rapid Equilibrium Random Mechanism

In Random mechanisms there is a random order in which substrates bind to the enzyme and in which the product unbind. The technique used to show strict convexity of $1/\tilde{f}$ does not apply directly to the complete rate function as it is produced by the King-Altman method. However, when we invoke a Rapid Equilibrium assumption in which all reactions are assumed to be fast except the step in which the enzyme bound by substrates is converted into the enzyme with products, the method does work. The rate equation of this mechanism is sometimes called convenience kinetics [6], and may be written as

$$f = \frac{\prod_{i=1}^{M} s_i - \frac{1}{K_{eq}} \prod_{j=1}^{K} p_j}{D},$$
$$D = \prod_{i=1}^{M}(1 + \tilde{K}_{s_i} s_i) + \prod_{i=1}^{K}(1 + \tilde{K}_{p_j} p_j) - 1,$$

for an $M-K$ Rapid Equilibrium Random Mechanism. This means the denominator D, among lots of other terms, includes the following

$$1, s_1, s_1 s_2, s_1 s_2, \dots, s_1 s_2 \cdots s_M, p_K, p_K p_{K-1}, \dots, p_K p_{K-1} \cdots p_1.$$

We have already shown in the proof of the Ordered mechanism that these terms yield strict convexity of $1/\tilde{f}$.

Free enzyme isomeration

Free enzyme isomeration means that the free enzyme form in the mechanism isomerizes. As an example, the Ordered Bi-Bi mechanism looks like

$$E \overset{\overset{A}{\downarrow}}{\longleftrightarrow} EA \overset{\overset{B}{\downarrow}}{\longleftrightarrow} EAB/FPQ \overset{\overset{P}{\uparrow}}{\longleftrightarrow} FQ \overset{\overset{Q}{\uparrow}}{\longleftrightarrow} F \longleftrightarrow E.$$

The rate equations for mechanism with isomerizing free enzymes are equal to the non-isomerizing rate equations with extra denominator terms [5]. Extra denominator terms can only give more directions of strict convexity of $1/\tilde{f}$. Therefore the proofs above for all non-isomerizing mechanisms also apply to the mechanisms with isomerizing free enzymes.

Activation

A kinetic mechanisms can also be influenced by an activator. A substance is an activator if it increases the reaction rate. An activator can theoretically effect any enzyme complex in the kinetic mechanism, just as an inhibitor. In all kinetics described above, the substrate is an activator, since we have $\partial v/\partial s > 0$. Linear activation means, equivalent to inhibition, that the activator activates the Michaelis-Menten constants in a linear way. For a reaction with linear activation, if the activating substance is absent, the reaction rate is zero. The effect of activation on the rate equation can be considered as the reciprocal of an inhibitor. Therefore all of the proofs above that we gave for inhibitors also apply to activators. This means that $1/\tilde{f}$ is strict convex with respect to the extra activator variable.

Substrate inhibition

Normally a substrate will work as an activator on a reaction, as we saw in the previous section. However, substrate inhibition is also possible. In an Ordered Bi-Bi reaction, substrate inhibition can occur in two different ways [4]. The first is unproductive enzyme binding, this means that the enzyme can also bind to the substrate to make an unproductive complex that cannot yield any product. With the second type of substrate inhibition, unproductive substrate binding, an extra substrate molecule S_1 interacts with the substrate-enzyme complex ES_1 to produce an unproductive complex S_1ES_1. Both types give linear inhibition. Therefore we have proved above that substrate inhibition on any of the mechanisms mentioned above yields a strict convex function $1/\tilde{f}$. Substrate inhibition yields linear uncompetitive inhibition for unproductive enzyme binding and linear competitive inhibition for unproductive substrate binding.

An exception to the rule: the Botts-Morales mechanism

The Botts-Morales modifier system is an example of a mechanism that can yield hyperbolic activation and inhibition. This means Michaelis Menten constants are modified in an hyperbolic way. Without activator there still could be catalytic activity of the enzyme. Also, saturation with inhibitor does not mean that there is no activity. The modifier mechanism for an irreversible Uni-Uni reaction has two ways to produce a product [4, §5.7.3]. The rate equation calculated with the King-Altman method is a very difficult expression with 12 numerator terms, and 15 denominator terms, including squared terms in both numerator and denominator [7]. However, if we assume that binding of modifier M to E and ES is at quasi-equilibrium [8, 9], the rate equation for this mechanism is:

$$f = \frac{(K_{(N,S)} + K_{(N,MS)}m + K_{(N,M2S)}m^2)s}{K_{(D,1)} + K_{(D,M)}m + K_{(D,S)}s + K_{(D,M2)}m^2 + K_{(D,MS)}ms + K_{(D,M2S)}m^2s}.$$

Rewriting this equation shows that f has the regular form of an irreversible reaction in s, if modifier M is a constant,

$$f = \frac{(K_{(N,S)} + K_{(N,MS)}m + K_{(N,M2S)}m^2)s}{(K_{(D,1)} + K_{(D,M)}m + K_{(D,M2)}m^2) + (K_{(D,S)} + K_{(D,MS)}m + K_{(D,M2S)}m^2)s}.$$

The inverse rate law is strict convex in s, but in m, $1/\tilde{f}$ exhibits concave behaviour.

Choosing suitable sensor concentrations

One of the main insights of the qORAC framework is that it can make predictions which intermediate metabolite concentrations may potentially act as sensors, relaying information to the gene network so that enzyme synthesis rates are changed. Within qORAC, the sensors should allow the construction of a predicted optimum $\boldsymbol{\xi}$. This means that it should be possible for sensor concentrations x_S, with S the index set of

sensors, to solve

$$\boldsymbol{\xi}_S = \boldsymbol{x}_S,$$
$$\frac{\partial O}{\partial \xi_i} = 0, \quad i \in I.$$

The real optima are defined by the external concentrations $\boldsymbol{x}_E$, and are found by minimising $O(\boldsymbol{x})$. These optima thus satisfy

$$\boldsymbol{\xi}_E = \boldsymbol{x}_E,$$
$$\frac{\partial O}{\partial \xi_i} = 0, \quad i \in I.$$

The Implicit Function Theorem gives explicit conditions for when the solution to a set of implicit equations in multiple variables may be locally written as functions of some of those variables. A simple example is the points (x, y) on a circle with radius 1 satisfying

$$x^2 + y^2 = 1.$$

Setting $f(x, y) = x^2 + y^2 - 1$, it is not possible to write the solutions to $f(x, y) = 0$ as the graph of one function $(x, g(x))$ for all x, nor as $(h(y), y)$, for all y. This is possible locally, however. For instance, as long as $\frac{\partial f}{\partial y} \neq 0$, $y = g(x)$ exists locally.

To apply the IFT to the implicit equations in the predicted optimum, we first introduce the following notation. Let R be the index set of all internal and external concentrations, so that $R = E \cup I$. Clearly, $S \subset I$. Denote by $R \backslash S$ the set of all indices in R which are not in S. The optima may now be written locally as functions of the sensor concentrations as long as the following condition is met,

$$\det \left(\frac{\partial}{\partial x_j} \frac{\partial O}{\partial x_i} \right)_{i \in I, j \in R \backslash S} \neq 0. \tag{1}$$

So we take the matrix of all partial derivatives of the optimum equations, delete the columns corresponding to a chosen set of sensors, and check if the determinant of this matrix is nonzero.

To be able to take the determinant, the above matrix needs to be square. From this we conclude that the number of deleted columns needs to be equal to the number of external sensors. The complete matrix with all partial derivatives of the optimum equations has $|E|$ more columns than rows, so we need to delete $|E|$ columns from it. Hence $|S| = |E|$.

The only steady state of a qORAC-controlled pathway is the optimum

We first consider a linear chain of reactions,

$$\underline{x}_0 \to x_1 \to x_2 \to \cdots x_N \to \underline{x}_{N+1} = 0, \tag{2}$$

where the metabolite x_i has dynamics

$$\dot{x}_i = v_i(e_i, x_{i-1}, x_i) - v_{i+1}(e_{i+1}, x_i, x_{i+1}), \quad i = 1, \ldots, N. \tag{3}$$

We assume reversible Michaelis-Menten kinetics,

$$v_i = e_i \frac{x_{i-1} - K_{eq}^i x_i}{a_i x_{i-1} + b_i x_i + c_i}, \quad i = 1, \ldots, N + 1. \tag{4}$$

for suitable positive constants K^i_{eq}, a_i, b_i and c_i. The external concentrations $\underline{x}_0$ and $\underline{x}_{N+1}$ are assumed fixed, as the underline indicates. We consider controlling the problem of controlling the pathway for changes in $\underline{x}_0$, and suppose that x_1 is the sensor.

The first theorem is supposedly well-known, but it has proved hard to find a good reference. We therefore prove it here, for completeness.

Theorem 2. *System* **(2)**–**(4)** *has a unique steady state for each choice of enzyme concentrations* $e_1, \dots, e_{N+1}$.

Proof. Write the system in the form

$$\dot{x}_i = F(x_{i-1}, x_i, x_{i+1}) \quad (i = 1, \dots, N), \quad x_0 = \underline{x}_0 > 0, x_{N+1} = \overline{x}_{N+1} > 0, \tag{5}$$

where

$$F_i = v_i - v_{i+1}. \tag{6}$$

Note that

$$\frac{\partial v_i}{\partial x_i} < 0 < \frac{\partial v_i}{\partial x_{i-1}} \tag{7}$$

for all nonnegative values of the concentrations. By the assumption on the ordering of the external concentrations, steady states are given by

$$v_1 = \cdots = v_{N+1} = \Psi > 0 \tag{8}$$

with Ψ to be determined. With F_i given by (6) there is precisely one such Ψ. To see this we vary Ψ from $\Psi = 0$ to at most $\Psi = \Psi_1 = v_1(\underline{x}_0, 0)$, and solve $\xi_1 = \xi_1(\underline{x}_0, \Psi)$, $\xi_2 = \xi_2(\underline{x}_0, \Psi)$, $\xi_3 = \xi_3(\underline{x}_0, \Psi)$ from

$$v_1(\underline{x}_0, \xi_1) = \Psi, \quad v_2(\xi_1, \xi_2) = \Psi, \quad v_3(\xi_2, \xi_3) = \Psi, \dots.$$

The sequence $\xi_1, \xi_2, \dots$ is well defined as long as in each step

$$\Psi < v_1(\underline{x}_0, 0) = \frac{\underline{x}_0}{g_1(\underline{x}_0, 0)}, \quad \Psi < v_2(\xi_1, 0) = \frac{\xi_1}{g_2(\xi_1, 0)}, \quad \Psi < v_3(\xi_2, 0) = \frac{\xi_2}{g_3(\xi_2, 0)}, \dots$$

For $\Psi = 0$ we have

$$\xi_1 = \frac{\underline{x}_0}{K^1_{eq}}, \quad \xi_2 = \frac{\xi_1}{K^2_{eq}} = \frac{\underline{x}_0}{K^1_{eq} K^2_{eq}}, \dots, \xi_{N+1} = \frac{\underline{x}_0}{K^1_{eq} \cdots K^{N+1}_{eq}} > \underline{x}_{N+1},$$

the latter inequality by assumption. Thus for small $\Psi > 0$ the sequence $\xi_1 > 0, \xi_2 > 0, \dots, \xi_{N+1} > 0$ is certainly well-defined by continuity and we have, using (7),

$$\frac{d\xi_1}{d\Psi} = \frac{1}{\frac{\partial v_1}{\partial x_1}(\underline{x}_0, \xi_1)} < 0, \quad \frac{d\xi_2}{d\Psi} = \frac{1 - \frac{\partial v_2}{\partial x_1}(\xi_1, \xi_2)\frac{d\xi_1}{d\Psi}}{\frac{\partial v_2}{\partial x_2}(\xi_1, \xi_2)} < 0, \quad \frac{d\xi_3}{d\Psi} = \frac{1 - \frac{\partial v_3}{\partial x_2}(\xi_2, \xi_3)\frac{d\xi_2}{d\Psi}}{\frac{\partial v_3}{\partial x_3}(\xi_2, \xi_3)} < 0, \dots$$

as long as this is the case. Clearly there will be a minimal Ψ_{N+1} such that all $\xi_1(\Psi), \dots, \xi_{N+1}(\Psi)$ are well defined as strictly positive numbers for all $\Psi < \Psi_{N+1}$. Note that Ψ_{N+1} exists because for $\Psi = \Psi_1 = v_1(\underline{x}_0, 0)$ we get $\xi_1 = 0$ in the first step.

In view of the monotonicity all $\xi_1(\Psi_{N+1}), \dots, \xi_{N+1}(\Psi_{N+1})$ exists as nonnegative numbers, and at least one of them must be zero, for otherwise Ψ_{N+1} is not minimal. Suppose some $i < N + 1$ is the largest i for which $\xi_i(\Psi_{N+1}) = 0$. Then we easily conclude that $\xi_{i+1}(\Psi_{N+1}) > 0$ is impossible for Ψ close to Ψ_{N+1}. Thus all $\xi_1(\Psi_{N+1}), \dots, \xi_N(\Psi_{N+1})$ are strictly positive and $\xi_{N+1}(\Psi_{N+1}) = 0$. Varying Ψ between 0 and Ψ_{N+1} there is a unique Ψ for which $\xi_{N+1}(\Psi_{N+1}) = \underline{x}_{N+1}$. This concludes the proof of the existence of a unique steady state. We did not actually use any specific properties of g_i, other than positivity and monotonicity. □

Theorem 3. *The qORAC-controlled linear chain as defined above has a unique steady state, which is the optimal steady state* $(\boldsymbol{x}^o, \boldsymbol{e}^o)$.

Proof. Let $\boldsymbol{x}^o$ be the unique optimal steady state corresponding to $\underline{x}_0$ and $\underline{x}_{N+1}$, with corresponding optimal enzyme concentrations e^o.

Let $(\boldsymbol{x}, \boldsymbol{\xi}, \boldsymbol{e}) \in \mathbb{R}^{n+2} \times \mathbb{R}^{n+2} \times \mathbb{R}^{N+1}$ be any steady state, and assume that $\boldsymbol{\xi}$ is a positive, strictly decreasing vector, so that the E_i functions are all well-defined and nonzero. We need to show that $\boldsymbol{\xi} = \boldsymbol{x} = \boldsymbol{x}^o$, so that immediately $\boldsymbol{e} = \boldsymbol{e}^o$.

Assume w.l.o.g. that x_1 is the sensor metabolite. We first show that $\boldsymbol{\xi} \equiv \boldsymbol{x}$ for indices 1 up to N.

The steady state equations in full are

$$e_i f_i(x_{i-1}, x_i) = e_{i+1} f_{i+1}(x_i, x_{i+1}), \quad i = 1, \ldots, N, \tag{9}$$

$$e_i = \frac{1/f_i(\xi_0, \ldots, \xi_{N+1})}{\sum_{k=1}^{N+1} 1/f_k(\xi_0, \ldots, \xi_{N+1})} \tag{10}$$

$$\frac{\partial O}{\partial x_i}(\xi_0, \ldots, \xi_{N+1}) = 0, \quad i = 1, \ldots, N, \tag{11}$$

$$x_0 = \underline{x}_0, \; x_{N+1} = \underline{x}_{N+1}, \tag{12}$$

$$\xi_1 = x_1. \tag{13}$$

We first substitute **(10)** into **(9)** so that we are left with **(11)**–**(13)** together with

$$\frac{f_1(x_0, x_1)}{f_1(\xi_0, \xi_1)} = \frac{f_2(x_1, x_2)}{f_2(\xi_1, \xi_2)} = \ldots = \frac{f_{N+1}(x_N, 0)}{f_{N+1}(\xi_N, \xi_{N+1})}, \tag{14}$$

The elements $x_2, \ldots, x_N$ satisfy

$$\frac{f_2(x_1, x_2)}{f_2(\xi_1, \xi_2)} = \ldots = \frac{f_N(x_{N-1}, x_N)}{f_N(\xi_{N-1}, \xi_N)} = \frac{f_{N+1}(x_N, 0)}{f_{N+1}(\xi_N, 0)}.$$

by **(14)**. Since $\xi_1 = x_1$ by **(13)**, this is equivalent to

$$\frac{f_2(\xi_1, x_2)}{f_2(\xi_1, \xi_2)} = \ldots = \frac{f_N(x_{N-1}, x_N)}{f_N(\xi_{N-1}, \xi_N)} = \frac{f_{N+1}(x_N, 0)}{f_{N+1}(\xi_N, 0)}. \tag{15}$$

These are steady state equations for a chain

$$\xi_1 \xrightarrow{v_2} x_2 \xrightarrow{v_3} \cdots \xrightarrow{v_{N-1}} x_{N-1} \xrightarrow{v_N} \xi_N \xrightarrow{v_{N+1}} \underline{x}_{N+1} = 0$$

in which the enzyme concentrations are set to $1/f_2(\xi_1, \xi_2), \ldots, 1/f_{N+1}(\xi_N, 0)$. By Theorem 2, for each set of enzyme concentrations the linear chain has a unique steady state. So $x_2, \ldots, x_N$ are uniquely determined. But, clearly, setting $x_2 = \xi_2, \; x_3 = \xi_3, \; \ldots x_N = \xi_N$ solves **(15)**. Hence, $\boldsymbol{x}$ and $\boldsymbol{\xi}$ must coincide for elements 2 to N.

Moreover, we deduce that

$$\frac{f_i(x_{i-1}, x_i)}{f_i(\xi_{i-1}, \xi_i)} = 1, \quad i = 1, \ldots, N+1.$$

The other elements of x can now be found in sequence. For instance, x_0 satisfies

$$f_1(x_0, \xi_1) = f_1(\xi_0, \xi_1).$$

Since $f_1(x_0, x_1)$ is monotone increasing in x_0, $x_0 = \xi_0$. We conclude that $\boldsymbol{x} = \boldsymbol{\xi}$.

Finally, we need to require for this steady state that $x_0 = \underline{x}_0$. But then also $\xi_0 = \underline{x}_0$, so that $\boldsymbol{\xi} = \boldsymbol{x}^o$, and $\boldsymbol{e} = \boldsymbol{e}^o$. Of course, this is not a real requirement, since our original optimum $\boldsymbol{x}^o$ satisfies these requirements trivially, and is hence the unique steady state. □

The argument in this proof relies on two statements that are true for a very wide class of pathways. First of all, for each choice of enzyme concentrations, there is a unique steady state for the metabolic concentrations (Theorem 2). This fixes the steady state optimum 'between' the sensor and the sink of the pathway. Second, one should be able to extrapolate from the sensor to the external concentrations (in the proof, x_0 is inferred from $x_1, \dots x_N$ by solving $f_1(x_0, \xi_1) = f_1(\xi_0, \xi_1)$. If the sensor is only a few reaction steps away from the external concentrations, this is a simple requirement. We summarise this in the following corollary.

Corollary 1. *Any qORAC-controlled pathway with the properties*

- *metabolic steady states are unique for each choice of enzyme concentrations;*
- *the sensors are connected to respective external concentration by a linear chain of reversible reactions;*

has a unique steady state, which is the optimum.

Numerical integration of the DAE

The code supplied with the paper allows the user to integrate the system as a system of Differential Algebraic Equations (DAEs) or as a set of Ordinary Differential Equations (ODEs). The code itself may be found at `http://www.few.vu.nl/~rplanque/Research/qORAC/`

For the implementation of qORAC as an ODE system, we recall the complete dynamical system with optimal control summarized in Box 1. This system is a DAE system of index 1. This means that by taking once the (implicit) time derivative of the algebraic equations for $\boldsymbol{x}$ and $\boldsymbol{\xi}$, explicit differential equations for $\dot{\boldsymbol{\xi}}$ may be given. Let $S \subset I$ be the index set of sensor metabolites $\boldsymbol{x}_S$. The algebraic equations for $\boldsymbol{\xi} = (\boldsymbol{\xi}_E, \boldsymbol{\xi}_I)$ and sensors $\boldsymbol{x}_S$ are

$$\frac{\partial O}{\partial \xi_i}(\boldsymbol{\xi}) = 0, \quad i \in I,$$
$$\boldsymbol{\xi}_S = \boldsymbol{x}_S.$$

Writing this system as $G(\boldsymbol{x}, \boldsymbol{\xi}) = 0$, and assuming that $\boldsymbol{\xi} = \boldsymbol{\xi}(t)$, we take the implicit derivative of these equations,

$$0 = D_x G \dot{\boldsymbol{x}} + D_\xi G \dot{\boldsymbol{\xi}},$$

where $D_x G$ is an $n \times n$ matrix consisting of zeros, but with a single 1 on each of the last $|S|$ rows, in position $s \in S$. Moreover,

$$D_\xi G = \begin{pmatrix} F \\ H \end{pmatrix}$$

is also an $n \times n$ matrix such that $F_{i,p} = \frac{\partial^2 O}{\partial \xi_i \partial \xi_p}$ for $i \in I, p = 1, \dots, n$ and

$$H_{l,p} = \begin{cases} -1 & p = s, \quad \text{for } l = 1, \dots, n - |I| \\ 0 & \text{otherwise.} \end{cases}$$

As a result, we find

$$\dot{\xi} = -D_\xi^{-1} G \begin{pmatrix} 0 \\ \vdots \\ 0 \\ \dot{x}_{k_1} \\ \dot{x}_{k_2} \\ \vdots \\ \dot{x}_{k_{|S|}} \end{pmatrix} \tag{16}$$

The system is thus of index 1 if and only if $D_\xi G$ is invertible. This is equivalent to the statement that the submatrix of F in which the columns corresponding to the sensors $\boldsymbol{\xi}_S$ are removed. And this is again equivalent to **(1)**, i.e., the fact that the optimum surface may be parametrized by the chosen sensors. The DAE system with chosen sensors is thus well-defined if and only if the DAE may be rewritten as a system of ODEs as detailed above, i.e. when it indeed is of index 1.

The resulting ODE system for $\dot{\boldsymbol{x}}, \dot{\boldsymbol{e}}, \dot{\boldsymbol{\xi}}$ is solved using standard `ode45` and `ode15s` Runge-Kutta solvers in Matlab.

For initial conditions, we refer to the code supplied in files `daes_CN.m` and `daes_CN_minimal_ICs.m`.

Numerical computation of input-output relations

Figure 2B in the main text contains an example of the optimal input-output relations (enzyme synthesis rates) for the concrete pathway described in the previous section. These functions may be reproduced using `sensor_optimum_enz_CN_newton.m`. The script computes optima for different sensor concentrations by varying the external concentration and solving the optimum equations $\frac{\partial O}{\partial x_i} = 0$ to find the corresponding optimum, and then plotting the resulting input-output relations against the sensor concentration values in the optima.

Additional illustrations of the qORAC framework

Reversing the predicted optimum

The sensor concentrations may be such that they predict an optimum in which the flow is opposite to the ones specified by the external concentrations. For instance, with high input concentration and low output concentration, the flow through a linear chain of reversible reactions is from input to output, and not reverse. If the sensor close to input has a low concentration, and the second sensor close to the output has a high concentration, the predicted optimum is one in which flow is from output to input. The predicted input and output concentrations are then low and high, respectively.

In such cases, the predicted optimum needs to ”reverse”, so that after some time the predicted external concentrations are in line with the real external concentrations. To do this, the predicted optimum needs to pass through a singular point, thermodynamic equilibrium. Exactly at this point, the Implicit Function Theorem breaks down. It is, however, easy to make a slight change to the optimum equations such that their solutions remain unchanged, but which avoids the singularity. An example is given in Figure S2. The code for this example may be found in `daes_linearchain_reversal.m`.

Full kinetic description of pathways

Pathway from Figure S1

We only specify the kinetics function and stoichiometry. The rest of the equations are the same as the first pathway, *mutatis mutandis*. The code for this pathway may be found in `daes_double_branced_sym.m`. The

equations for the pathway are

$$\begin{aligned}
\dot{x}_1 &= 0,\\
\dot{x}_2 &= v_1 - v_3,\\
\dot{x}_3 &= 0,\\
\dot{x}_4 &= v_2 - v_3,\\
\dot{x}_5 &= v_3 - v_4,\\
\dot{x}_6 &= v_4 - v_5,\\
\dot{x}_7 &= v_5 - v_6,\\
\dot{x}_8 &= 0,\\
\dot{x}_9 &= v_3 - v_7,\\
\dot{x}_{10} &= v_7 - v_8,\\
\dot{x}_{11} &= v_8 - v_9,\\
\dot{x}_{12} &= 0.
\end{aligned}$$

with

$$\begin{aligned}
f_1 &= \frac{x_1 - x_2}{5.0x_1 + 4.0x_2 + 3.0},\\
f_2 &= \frac{x_3 - x_4}{x_3 + 6.0x_4 + 1.0},\\
f_3 &= \frac{x_2x_4 - x_5x_9}{3.0x_2 + 5.0x_4 + x_5 + 6.0x_9 + 3.0},\\
f_4 &= \frac{x_5 - x_6}{3.0x_5 + x_6 + 3.0},\\
f_5 &= \frac{x_6 - x_7}{2.0x_6 + 3.0x_7 + x_{10} + 1.0},\\
f_6 &= \frac{x_7 - x_8}{3.0x_7 + 4.0x_8 + 2.0},\\
f_7 &= \frac{x_9 - x_{10}}{2.0x_9 + x_{10} + 3.0},\\
f_8 &= \frac{x_{10} - x_{11}}{2.0x_5 + x_{10} + 3.0x_{11} + 1.0},\\
f_9 &= \frac{x_{11} - x_{12}}{3.0x_{11} + 3.0x_{12} + 3.0}.
\end{aligned}$$

Note that x_1, x_3, x_8 and x_{12} are fixed external concentrations.

Pathway from Figures 2 and S3

We only specify the kinetics function and stoichiometry. The rest of the equations are the same as the first pathway, *mutatis mutandis*. The code for this pathway may be found in `daes_linearchain_reversal.m`. The pathway is a linear chain of reversible Michaelis-Menten kinetics reactions, and is given by

$$\dot{x}_i = v_{i-1} - v_i, \quad i = 2, \ldots, 6,$$

(x_1 is again fixed), and

$$v_i = e_i \frac{x_i - x_{i-1}}{A_i + B_i x_{i-1} + C_i}, \quad i = 1, \ldots, 7,$$

The exact parameters may be found in the matlab file `daes_linearchain_reversal.m` (Figure S3), and `daes_CN_minimal_ICs` (Figure 2).

References

1. Noor E, Flamholz A, Bar-Even A, Davidi D, Milo R, Liebermeister W. The Protein Cost of Metabolic Fluxes: Prediction from Enzymatic Rate Laws and Cost Minimization. PLoS Comp Biol. 2016;12(11):e1005167.

2. Botts J, Morales M. Analytical description of the effects of modifiers and of enzyme multivalency upon the steady state catalyzed reaction rate. J Trans Faraday Soc. 1953;49:696–707.

3. King EL, Altman C. A Schematic Method of Deriving the Rate Laws for Enzyme-Catalyzed Reactions. J Phys Chem. 1956;60:1375–1378.

4. Cornish-Bowden A. Fundamentals of Enzyme Kinetics. 4th ed. Wiley-Blackwell; 2004.

5. Cleland WW. The kinetics of enzyme-catalyzed reactions with two or more substrates or products: I. Nomenclature and rate equations. Biochim Biophys Acta. 1963;67:104–137.

6. Liebermeister W, Klipp E. Bringing metabolic networks to life: convenience rate law and thermodynamic constraints. Theor Biol Medical Modelling. 2006;3:42.

7. Kuzmic P. Program DYNAFIT for the Analysis of Enzyme Kinetic Data: Application to HIV Proteinase. Anal Biochem. 1996;237:260–273. (Online King–Altman tool: http://www.biokin.com/cgi–bin/king–altman/kingalt.cgi).

8. Segel IH, Martin RL. The general modifier (”allosteric”) unireactant enzyme mechanism: redundant conditions for reduction of the steady state velocity equation to one that is first degree in substrate and effector. J Theor Biology. 1988;135:445–453.

9. Topham CM, Brocklehurst K. In defence of the general validity of the Cha method of deriving rate equations. The importance of explicit recognition of the thermodynamic box in enzyme kinetics. Biochem J. 1992;282:261–265.
