# Supplementary figures and images for: Maintaining maximal metabolic flux by gene expression control
Source: PLoS Comput Biol. 2018 Sep 20;14(9):e1006412. doi: 10.1371/journal.pcbi.1006412 (PMC6168163; doi:10.1371/journal.pcbi.1006412)

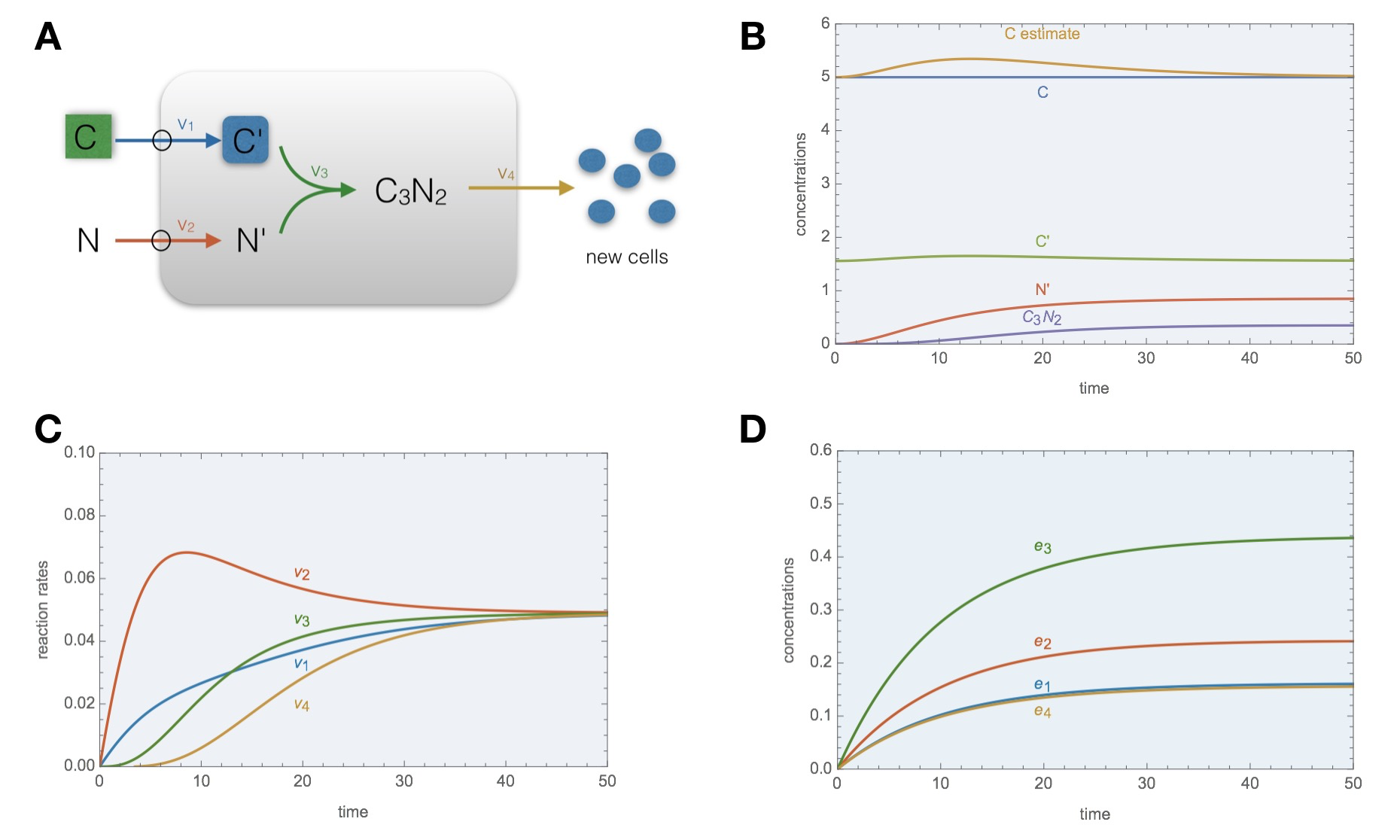

Supplement: S1 Fig — The pathway is still steered to the optimal specific flux steady state. A: the pathway; B: metabolite concentrations over time (all except orange), and predicted optimal external metabolite concentration (orange); C: reaction fluxes over time; D: enzyme concentrations over time. See Box 2 in main text for details of the pathway, and the matlab file daes_CN_minimal_ICs.m for the code. (TIF) [file pcbi.1006412.s002.tif]

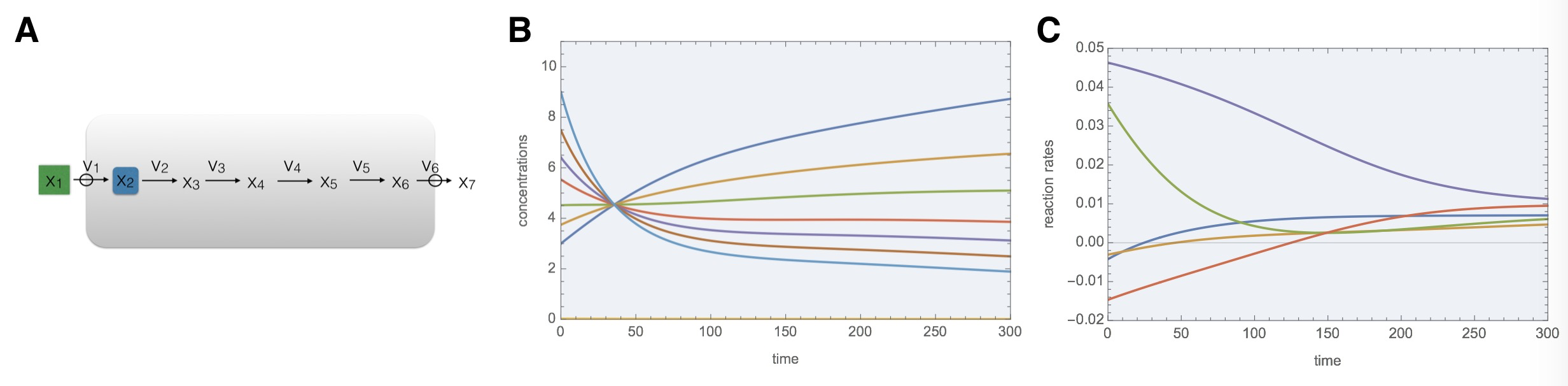

Supplement: S2 Fig — Although the requirements for sensor control are not upheld in this point, the predicted optimum moves smoothly through this singular points and the system adapts as it should. A: linear chain pathway, with external conditions such that flow is initially from x1 to x7; B: dynamics for the predicted optimal metabolite concentrations (ξ). The intersection point of all the curves is thermodynamic equilibrium; C: reaction flux dynamics. Note that the fluxes do not pass through v1 = ⋯ = v6 = 0. Three do, and the others do not, in this example. See SI text for details of the pathway, and the matlab file daes_linearchain_reversal.m for the code. (TIF) [file pcbi.1006412.s003.tif]

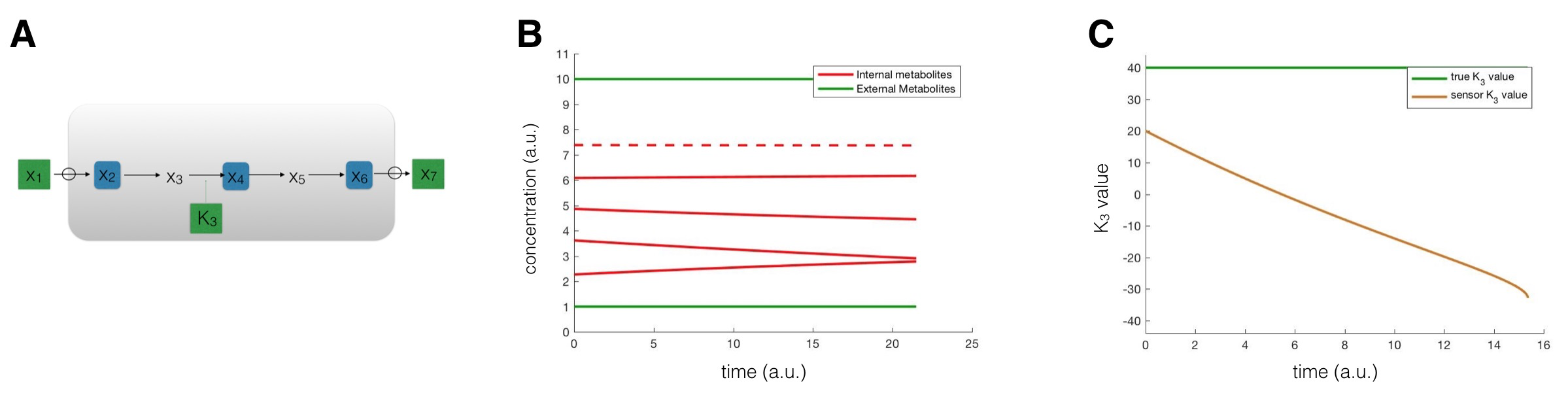

Supplement: S3 Fig — A: The pathway, which is identical to that in Fig 5 in the main text—only the choice of sensors (in blue) is different. Sensor x3 is swapped with x4. B/C: The dynamics of metabolites (B) and predicted K3 values (C) do start to change. However, the dynamics converge to a singular point, and the dynamical system can not continue. This second choice of sensors does not yield a gene expression control system which steers the pathway to optimal specific flux. See matlab code daes_extra_param_wrong.m for the code. (TIF) [file pcbi.1006412.s004.tif]

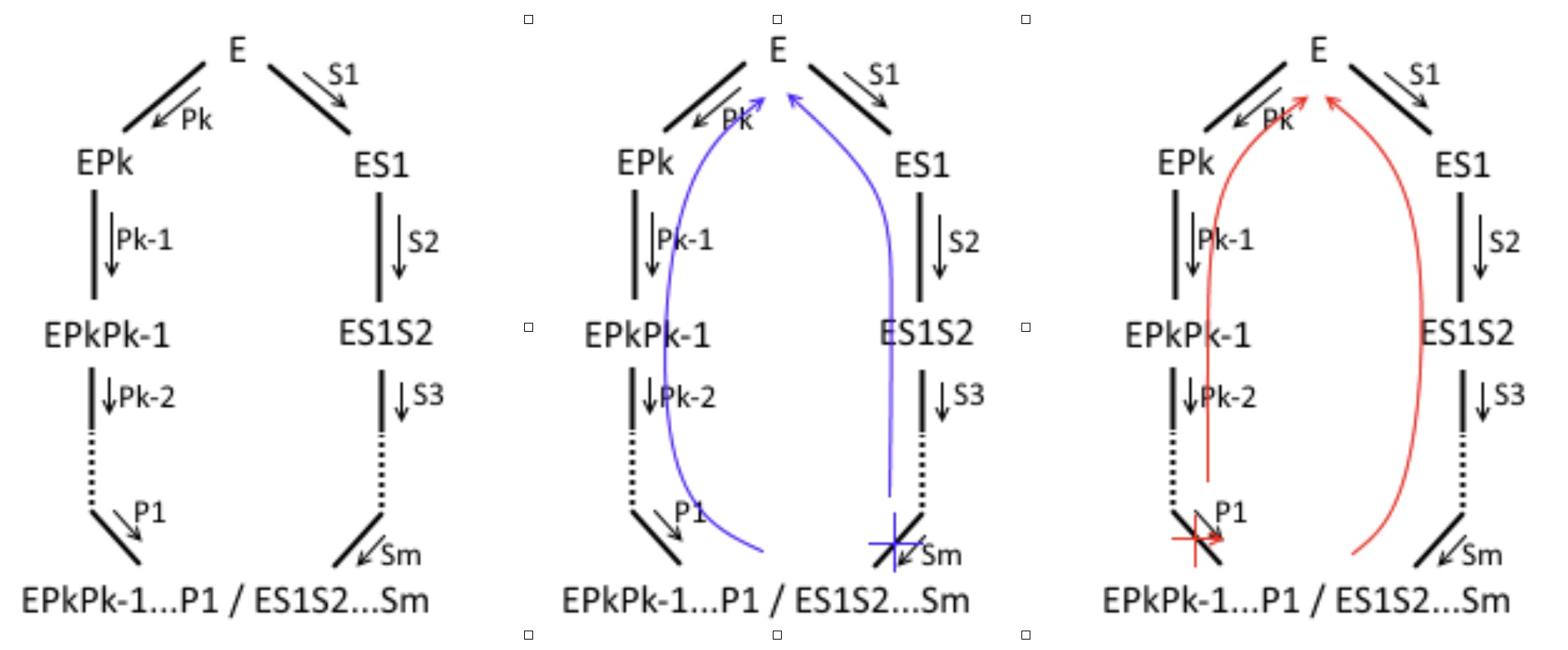

Supplement: S4 Fig — The left one shows the master pattern, the middle and right figure show two alternative patterns that yield the constant term in the denominator of f. (TIF) [file pcbi.1006412.s005.tif]
